# DNA Nanostructures for Rational Regulation of Cellular Organelles

**DOI:** 10.1021/jacsau.5c00117

**Published:** 2025-03-26

**Authors:** Petra Elblová, Judita Anthi, Minghui Liu, Mariia Lunova, Milan Jirsa, Nicholas Stephanopoulos, Oleg Lunov

**Affiliations:** † FZU - Institute of Physics of the Czech Academy of Sciences, 182 21 Prague, Czech Republic; ‡ Faculty of Mathematics and Physics, Charles University, Ke Karlovu 3, 121 16 Prague 2, Czech Republic; § School of Molecular Sciences, Arizona State University, Tempe, Arizona 85287, United States; ∥ Biodesign Center for Molecular Design and Biomimetics, Arizona State University, Tempe, Arizona 85281, United States; ⊥ Institute for Clinical & Experimental Medicine (IKEM), 14 021 Prague, Czech Republic

**Keywords:** DNA nanotechnology, DNA nanostructures, Organelle interference, Cellular functions, Self-assembly, Nanomedicine

## Abstract

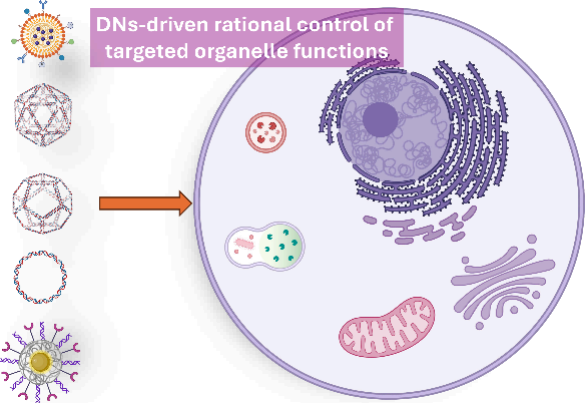

DNA nanotechnology has revolutionized materials science and biomedicine by enabling precise manipulation of matter at the nanoscale. DNA nanostructures (DNs) in particular represent a promising frontier for targeted therapeutics. Engineered DNs offer unprecedented molecular programmability, biocompatibility, and structural versatility, making them ideal candidates for advanced drug delivery, organelle regulation, and cellular function modulation. This Perspective explores the emerging role of DNs in modulating cellular behavior through organelle-targeted interventions. We highlight current advances in nuclear, mitochondrial, and lysosomal targeting, showcasing applications ranging from gene delivery to cancer therapeutics. For instance, DNs have enabled precision mitochondrial disruption in cancer cells, lysosomal pH modulation to enhance gene silencing, and nuclear delivery of gene-editing templates. While DNs hold immense promise for advancing nanomedicine, outstanding challenges include optimizing biological interactions and addressing safety concerns. This Perspective highlights the current potential of DNs for rational control of targeted organelles, which could lead to novel therapeutic strategies and advancement of precision nanomedicines in the future.

## 1 Introduction

Advancements in the manipulation, organization, and utilization of matter at the nanoscale have driven the rapid growth of nanotechnological products in recent years. Among the various branches of nanotechnology, the biomedical application of nanomaterials stands out as a particularly promising area, offering innovative solutions for diagnostics, therapeutic delivery, and disease management.^1−3^ Significant advancements in the biomedical applications of engineered nanoparticles have been achieved, enhancing therapeutic outcomes through increased drug bioavailability, precise targeting of diseased tissues, and a reduction in off-target effects, all of which mitigate the adverse side effects commonly associated with conventional drug therapies.^4−6^ By 2022, the U.S. Food and Drug Administration (FDA) had approved 23 nanoparticle-based medicine products, with a steadily growing pipeline of candidates undergoing clinical trials.^6−8^

The majority of FDA-approved nanobased medicines revolve around well-established nanoparticle systems, such as micelles, liposomes, polymeric nanoparticles, and inorganic nanoparticles.^6−8^ While these systems have proven effective, they are still limited by heterogeneity in particle size and shape, chemical and physical instability, inability to precisely control ligand spacing and stoichiometry, and potential cytotoxicity. These limitations present challenges, preventing broader utility and long-term applicability of nanobased medicines.^8−12^ Furthermore, the poor clinical performance of certain nanoparticle-based medicines has already led to the withdrawal of several nanodrugs.^8,12−17^ Consequently, the number of FDA-approved nanomedicines declined from over 50 in 2016 to just 23 by 2022.^7,8,18,19^

Current advances in molecular self-assembly, specifically in nucleic acid nanotechnology, have opened exciting new possibilities, particularly through the exploration of programmable biopolymeric materials like DNA as nanocarriers.^20−25^ DNA is inherently biocompatible, can be precisely programmable at molecular level, and is structurally versatile. These properties offer a unique foundation for the design of highly controlled, nanoscale drug delivery systems based on DNA.^24,26,27^ Although still in the early stages of development, DNA nanocarriers have shown tremendous potential for enhancing drug delivery mechanisms, enabling precise targeting, and minimizing adverse effects.^28−31^ DNA nanostructures (DNs) are particularly valuable for studying and interacting with biological systems due to their precise ability to position bioactive molecules at the nanometer scale.^32,33^ Specifically, functionalized DNA origami have been instrumental in uncovering the nanoscale mechanisms underlying immune receptor activation.^34−37^ Additionally, DNs have proven highly effective in investigating enzymatic processes,^38,39^ or to determine the spatial tolerances of antibody–antigen interactions at the nanoscale.^40^ Finally, nanometer-precise tuning of CpG oligonucleotide spacing in DNs significantly enhanced the potency of antitumor vaccines by boosting dendritic cell (DC) and natural killer cell activation *in vivo*.^41^

It is worth noting that nucleic-acid–based therapeutics, have demonstrated significant therapeutic potential, resulting in the recent approval of 20 products by the FDA and the European Medicines Agency (EMA). These therapeutics include chemically modified antisense oligonucleotides, N-acetylgalactosamine ligand-modified short interfering RNA conjugates, lipid nanoparticles, and adeno-associated virus vectors.^42^ However, despite significant progress in the design of innovative nucleic acid–based nanostructures and the evaluation of their therapeutic efficacy in rodent models,^24,26,27,29^ clinical application of these nanostructures remains unrealized.^26,29,30,43^ At present, the most advanced clinical trials for these technologies are in Phase 0/1, underscoring their early stage of development for human use.^30^ A major barrier to progress may be our still limited understanding of how nucleic acid nanostructures interact with cells at disease sites and the biological responses triggered by their internalization.^26,29,30,43^ Overcoming this challenge requires rigorous and comprehensive studies of *in vivo* interactions between nucleic acid nanostructures and target cells.^30,43^ Such research holds the potential to uncover fundamental insights into the mechanisms of action of these nanostructures, and to identify strategies for enhancing their therapeutic efficacy. By expanding our knowledge of cell-nanostructure interactions, we can facilitate the development of more effective and clinically viable nucleic acid nanostructures, ultimately advancing their application in precision medicine.

In recent years, the targeted regulation of organelles has emerged as a transformative strategy for modulating cellular behavior and dictating cell fate, offering exciting possibilities for disease intervention and treatment.^44−47^ This approach has garnered widespread attention due to its ability to precisely influence fundamental cellular processes, paving the way for innovative therapeutic solutions.^48^ Organelles within a cell do not function in isolation; rather, they operate in close coordination with one another to execute a wide range of key cellular processes.^48−51^ The disruption or deliberate regulation of specific organelles can significantly impact cellular homeostasis, often resulting in impaired cell function or alterations in cellular behavior.^48−51^ In fact, dysregulated organelles have been found to play a key role in the development and progression of various diseases and pathological conditions.^48^

As a result, organelle-targeting strategies that focus on selectively disrupting or modulating specific organelles have emerged as a highly promising area of research.^48−51^ These strategies hold particular significance in the field of precision medicine, especially in the context of cancer treatment, where precise targeting of organelles could selectively eliminate cancer cells while minimizing harm to healthy tissue.^48−51^ For example, enzymatically generated supramolecular assemblies of short peptides have been designed to specifically target the endoplasmic reticulum, leading to selective cancer cell death.^52^ Additionally, custom-engineered metal complexes have been developed to disrupt lysosomal integrity, producing therapeutic effects by inducing cellular damage in diseased cells.^53,54^

Recent studies have also demonstrated that DNs can serve as highly versatile platforms for precise control over cellular functions by targeting and regulating organelles through rational design, via so-called “organelle interference”.^55−59^ We believe that this innovative approach signifies a paradigm shift, introducing a new class of nanocarriers that could revolutionize the field of nanomedicine and expand the therapeutic landscape.

While several reviews have examined the interactions between DNA nanostructures and cells, these discussions are typically approached from a physicochemical standpoint, or fail to address the critical issue of organelle interference induced by DNs.^24,26−30^ To date, there has been no comprehensive and critical analysis of how DNs modulate cellular functions through targeted organelle regulation. Moreover, there is a notable lack of critical evaluation regarding the cellular models employed in these studies, and no systematic categorization of the resulting biological outcomes. Thus, in this Perspective, we aim to organize and contextualize the existing knowledge on DN-based modulation of cellular behavior via organelle interference. Our goal is to present a structured framework that highlights DNA-based nanomaterials as versatile platforms for the rational control of organelles, setting a new paradigm for the development of nanomaterial systems capable of precisely guiding cellular functions. Furthermore, we provide a very brief overview of the fundamental design strategies for DNA nanostructures used in biomedical applications, highlighting examples of specific organelle delivery and interactions. For a more in-depth discussion of these topics, readers are directed to comprehensive reviews available in the literature.^24,60−63^

## 2 Design of DNA Nanostructures for Organelle Interference

DNA nanotechnology initially centered on the discovery and rational design of structural motifs, such as DNA tiles, junctions, and DNA origami, which serve as foundational units for creating more complex architectures.^24,27,63^ These early studies created the groundwork for the field’s rapid growth, particularly since the early 2000s, enabling researchers to construct highly sophisticated nanoscale systems with programmable properties.^24,27,63^ Seeman’s pioneering concept of designing complex DNA-based structures was experimentally validated shortly after its proposal, marking a critical milestone in structural DNA nanotechnology.^63,64^ This foundational work set the stage for the creation of branched DNA motifs, which enabled the construction of various covalently closed, discrete 3D objects, such as a DNA cubes and tetrahedra.^24,27,63^ Building on these advances, modular DNA tiles were introduced as versatile nanoscale building blocks for constructing complex, higher-order assemblies. These included discrete nanocages, nanotubes, 2D arrays, and even macroscopic crystals.^24,27,63^ Overall, recent advancements in the synthesis and design of DNA nanostructures have enabled the creation of nanocarriers with precisely defined geometries and highly programmable molecular interactions. These nanocarriers can be loaded with specific drugs, and functionalized with tailored chemical motifs to selectively target specific biomolecules.^24,27,63^ In particular, DNA origami represents a versatile and powerful technique that enables the construction of highly precise nanoscale structures by folding long single-stranded DNA scaffolds with short, complementary staple strands.^24,65^ This method allows for the design and fabrication of complex 2D and 3D nanostructures with remarkable near-atomic precision.^24,65^ The use of computer-aided design tools has revolutionized the process, providing the ability to generate detailed DNA origami blueprints that guide the folding process.^24,65^ Recent advancements in scaffold synthesis have enhanced the versatility of DNA origami, enabling the creation of scaffolds with custom lengths and sequences tailored to specific applications.^66^ The folding process itself is highly cooperative, driven by the DNA sequence, and typically follows efficient folding pathways that lead to well-defined, stable structures.^67^

### 2.1 Structural Advantages of DNs

DNs have found applications across a wide range of fields, including nanofabrication, targeted drug delivery, and biosensing. Specifically, DNs offer exciting opportunities for targeting intracellular organelles and enabling the controlled manipulation of their functions, which opens new avenues for both research and practical applications.^24,26−30,65,68,69^ Other synthetic nanomaterials also exhibit excellent biocompatibility, can be functionalized with ligands targeting various organelles, and can be synthesized in diverse sizes and shapes.^70,71^ However, the programmability of DNs, particularly their size, shape, aspect ratio, and surface chemistry, provides unique advantages over other nanomaterials in biomedical applications.^24,26−30,65,68^

For example, the size and shape of nanoparticles significantly influence their behavior in biological systems.^72^ The optimal size for biomedical applications typically ranges from 1 to 100 nm, and impacts factors like cellular uptake, biodistribution, and circulation half-life.^73^ Nanoparticles in the 10–60 nm range show the most efficient cellular uptake, while those larger than 100 nm can penetrate leaky tumor vasculature but not dense collagen matrix.^74−76^ The shape of nanoparticles also plays a crucial role in cellular interactions and pharmacokinetics.^77^ For instance, cylindrical filomicelles tend to demonstrate longer blood circulation times compared to spherical nanoparticles.^77^

While various methods for nanoparticle synthesis exist, conventional approaches often struggle to control size and shape precisely.^78−80^ Mechanical agitation during synthesis, for example, can induce aggregation and alter particle morphology.^81^ This lack of consistency can lead to heterogeneity in nanoparticle size and shape, which significantly affects the performance and reliability of biomedical applications, particularly in drug delivery systems.^82,83^ Variations in size and shape in turn can influence cellular uptake, biodistribution, and drug release kinetics, making it challenging to optimize and translate these systems clinically.^80^ In contrast, the nanoscale addressability of DNs allows for more precise and controllable design, making them ideal candidates for the synthesis of customized nanoscale drug delivery systems and other biomedical applications.^24,26−30,65,68^ This unique level of control over size, shape, and surface chemistry provides significant advantages in optimizing performance and ensuring consistency across potential therapeutic batches.^24,26−30,65,68^

Another distinct advantage of DNs is the precise spatial arrangement of ligands and payloads, including their specific quantities, stoichiometries, spacing, and overall spatial patterns.^24,26−30,61,62,65,68^ Studies have demonstrated that controlled spacing and patterning of ligands can significantly enhance target binding affinity and signal activation potency.^37,84^ For example, DNA nanoscaffolds have been used to determine optimal ligand distances, such as 7 nm for effective activation of Toll-like Receptor 9.^85^ DNA origami-based designs that replicate viral surface patterns have shown improved sensing and inhibition capabilities.^86^ Furthermore, spatially patterned heteromultivalent nanostructures exhibit markedly higher affinity compared to their unpatterned counterparts.^87^

The importance of interligand distances in multivalent binding was further highlighted in studies using DNA nanoscaffolds.^88^ Specifically, the positioning of ligands on DNA frameworks was shown to influence targeting performance, with larger ligand distances generally yielding optimal effects.^89^ DNA origami-based nanoarrays have also been found to enhance cell binding affinity by matching the spatial distribution of membrane proteins.^90^ Research has revealed that the spatial patterning of oligonucleotides on nanoparticles can drastically improve binding affinity, with correctly patterned particles demonstrating up to 23 orders of magnitude higher affinity than those with incorrect patterns.^87^ Additionally, DNA tetrahedra have been utilized for targeted siRNA delivery to tumors, with a minimum of three folate molecules per nanoparticle being necessary for optimal delivery.^91^ Recent studies have further emphasized that ligand spacing and arrangement on DNA frameworks can significantly affect targeting efficiency and bioavailability, both *in vitro* and *in vivo*.^89,92^

To summarize, DNs offer several key structural advantages that enhance their effectiveness in organelle targeting. First, their design flexibility provides precise control over size, shape, and functionality, allowing for the integration of multiple functional components (e.g., targeting ligands, sensing modules, and therapeutic payloads) into complex, multifunctional devices.^93^ These can be tailored to meet specific targeting and functional requirements of individual organelles.^93^ Second, the incorporation of various functional modules enables DNs to perform a range of tasks, from targeting specific organelles to detecting cellular conditions and delivering therapeutic payloads.^24,26−30,65,68,93^ This multifunctionality makes them highly versatile for addressing complex biological processes. Third, DNs allow for precise molecular recognition, such as sequence-specific DNA hybridization.^94^ This capability enables highly accurate targeting, with the potential for interacting with specific genomic regions or cellular components, further enhancing the precision of their delivery and function.^24,26−30,65,68,93,94^

It is important to note that despite their potential, DNs face several limitations when it comes to biomedical applications, which currently hinder their clinical translation. These challenges include limited intracellular stability, potential immune responses, and poorly understood biodistribution profiles.^26,95^ Enzymatic degradation and low cellular uptake further complicate their effective use in therapeutic contexts.^96,97^ Additionally, issues related to production scale and high synthesis costs remain significant barriers to widespread clinical adoption.^24,26−30,61,62,65,68^ Despite the limitations mentioned above, DNs represent an excellent platform for the *rational regulation of organelle functions* through precise targeting. This approach can be understood as the *controlled manipulation of subcellular processes using engineered nanomaterials*. For instance, nanoparticles can induce size-dependent, reversible mitochondrial fission without causing cell injury, by enhancing lysosome-mitochondria interactions.^98^ Additionally, nanoparticles can regulate autophagy through mechanisms involving organelle damage, oxidative stress, and signaling pathways.^99^ Moreover, synthetic nanoassemblies can be designed to interfere with the natural architecture of organelles, offering potential for precision therapeutics.^51^

### 2.2 DNA-Based Nanostructures for Organelle Targeting

It is important to note that some organelles are easier to target than others due to their unique structural features, accessibility, and functional roles in cellular processes.^48^ For example, organelles like the nucleus and mitochondria have distinct membrane compositions that can be exploited for targeted delivery. The nuclear envelope, for instance, can be targeted by nuclear localization sequences (NLSs) that facilitate the entry of specific proteins and drugs.^48,100,101^ Similarly, some organelles possess unique surface markers or receptors that can be targeted by ligands or antibodies, enhancing the specificity of drug delivery. The Golgi apparatus can be targeted using specific peptides that recognize its unique membrane proteins,^48,100,101^ and organelles like the endoplasmic reticulum (ER), which are involved in protein folding and stress responses, are often targeted in cancer therapy to induce stress responses and trigger cell death.^48,100,101^

Organelles located closer to the cell membrane, such as lysosomes, may also be more accessible for drug delivery systems compared to those deep within the cytoplasm, like the nucleus.^48,100,101^ However, the structure and function of organelles can vary significantly across different cell types, complicating the development of universal targeting strategies. For instance, the sarcoplasmic reticulum in muscle cells has unique features compared to the ER in other cell types, which makes creation of a universal targeting approach challenging.^48,100,101^ Additionally, organelles like the ER and Golgi apparatus share membrane characteristics with the cell membrane, which makes it difficult to distinguish them for precise delivery. The dynamic nature of organelles (such as their fragmentation and reassembly during processes like cell division or stress responses) further complicates targeting strategies.^48,100,101^

The above-described unique advantages of DNs enable their potential for organelle targeting include structural adaptability, molecular recognition, and modular functionality that allows specific targeting across different cellular compartments. We believe that structural advantages of DNs in design flexibility and precise spatial arrangement of ligands and payloads create a foundation to design DN platforms that may overcome current challenges in organelle targeting. For example, custom-designed DNA devices combine structural flexibility, environmental responsiveness, and modular functionality to address diverse targeting challenges.^102^ Current research indicates that organelles such as the nucleus, mitochondria, and lysosomes can already benefit from existing DNA-based nanoformulations. For instance, mitochondrial targeting has been improved through peptide and lipid modifications that enable selective delivery and metabolic interference.^103^ Lysosomes are targeted using pH-responsive dynamic assemblies that transform from nanoparticles to hydrogels in acidic conditions, facilitating controlled activation.^102^ For nuclear targeting, DNA nanostructures have enabled enhanced delivery of anticancer drugs, gene regulatory components, and clustered regularly interspaced short palindromic repeats (CRISPR) elements for genomic integration.^104^ Furthermore, ER targeting is feasible using ligand-based strategies that overcome membrane barriers and promote efficient protein degradation.^105^ Additional features of DNs include the precise molecular recognition offered by sequence-specific hybridization and the integration of multiple functional modules (e.g., targeting ligands, sensing elements, and therapeutic payloads) within a single nanostructure.^94^ These characteristics enable DNA nanodevices to effectively target and operate across a variety of organelles, including mitochondria, the nucleus, lysosomes, the ER, and others.^31,106−108^

Generally, to achieve precise organelle targeting, engineered nanomaterials can be designed to incorporate specific moieties that guide them to the desired cellular organelle. For instance, ligands targeting the nucleus, such as nuclear localization signal peptides, can be integrated into the design to facilitate transport across the nuclear envelope.^109−111^ NLSs—such as those derived from the SV40 T antigen, adenovirus, transactivator of transcription (TAT) peptide, NF-κB, or the KRRRR sequence—are among the most widely used and well-established ligands for facilitating accumulation in the nucleus.^109−112^ Similarly, mitochondria-targeting strategies often use moieties that are both positively charged and lipophilic, enabling them to traverse the mitochondrial membrane’s electrochemical gradient and localize within the organelle.^110,111,113^

**Figure 1:**
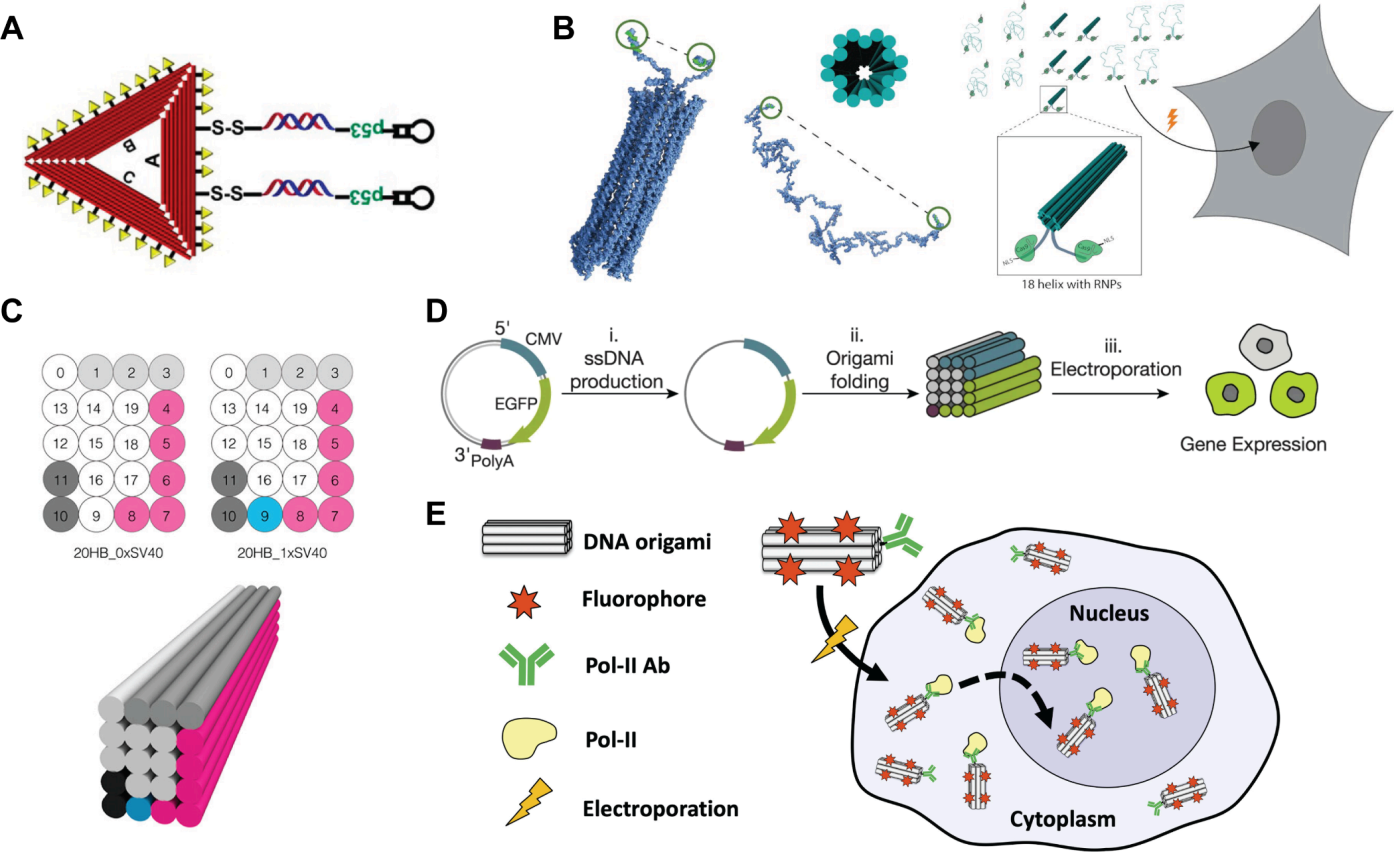
DNs for nucleic delivery. (A) Schematic of DNA-based nanocarrier for efficient gene delivery and combined cancer therapy. Reproduced from ref (114). Copyright 2018 American Chemical Society. (B) Schematic of CRISPR-Cas9 binding to the ends of unstructured, looped and 18-helix nanostructure templates. CRISPR-Cas9 carries NLS to enter the nucleus upon electroporation. Reproduced from ref (115). CC BY-NC 4.0. (C) Schematic cross section of helices (0–19) for the 20HB structures displaying sequences of interest in the exterior helices. Cylindrical model of the used DNA origami structure, a 20-helix bundle (20HB). Reproduced from ref (116). CC BY 4.0. (D) Folding and expressing genes from origami structures. Schematics of the overall workflow: ssDNA is produced from plasmid DNA via phagemids (i) and then folded into 20 helix bundle (20HB) DNA origami objects (ii); objects were delivered to cells, and gene expression from the origami structure was assessed by detection of positive fluorescence read-out (iii). CMV promoter sequence is shown in blue, gene encoding for enhanced green fluorescent protein (EGFP) in green, and the bGH polyadenylation signal (polyA) in purple. Reproduced from ref (117). CC BY 4.0. (E) Concept for piggybacking DNA nanostructures into the nucleus. DNA nanostructures functionalized with RNA Pol II-targeting antibodies and eight Cy5 fluorophores are electroporated into cells, bound to Pol II, and then imported, or piggybacked, into the nucleus. Reproduced from ref (118). CC BY-NC 4.0.

We summarize current studies on the utilization of DNA nanotechnology for targeted organelle manipulation in Table 1. Unlike other reviews, which systematically evaluate subcellular organelle targeting with a primary focus on the properties and synthesis of DNA nanomaterials,^51,63,119−121^ our work is organized based on the specific targeted organelles. We place particular focus on the strategies employed for organelle targeting (Table 1). Interestingly, nuclear targeting strategies for DNs exhibit significant variability, ranging from the use of conventional NLS signals, such as SV40,^116^ to more advanced approaches (Table 1 and Figure 1). These include the incorporation of CRISPR-Cas9 ribonucleoprotein binding sites^115^ and the use of RNA polymerase II (Pol II)-targeting antibodies to facilitate active nuclear import.^118^ It is evident that the main strategies for targeting the nucleus involve the use of NLS and antibody conjugation (Table 1 and Figure 1). The efficacy of these targeting methods is largely determined by the ability of DNs to cross the nuclear pore complex and by their DNA compaction.^115,122^ Key limiting factors for effective nuclear delivery include the size of the DNs and the potential for nuclear exclusion.^118^ For example, it has been shown that the 26HB structure (∼90 nm in length and ∼ 10–15 nm in cross-sectional dimensions) is unable to enter the nucleus,^118^ suggesting that the size of the nuclear pore complex may be a limiting factor. The central pore of the nuclear pore complex in human cells, for instance, has a diameter of approximately 110 nm,^123^ which means that larger DNs may face challenges in penetrating the nucleus. Furthermore, a major hurdle for large DNs in nuclear entry is cytoplasmic aggregation,^118^ which can hinder their effective delivery to the nucleus.

**Table 1:** Design of DNs for Organelle Targeting^a^

organelle	DN format	DN structural features	organelle targeting strategy	size	zeta potential	ref
Nucleus	18HB	Helix bundles	CRISPR-Cas9 ribonucleoprotein binding sites	29 ± 10 nm	Not reported	(115)
	20HB	Helix bundles	SV40 DTS	∼100 nm	Not reported	(116)
	20HB	Helix bundles	Electroporation and inclusion of CMV promoter sequence	20HB: 69 nm	Not reported	(117)
	12HB			12HB: 114 nm		
	32HB			32HB: 42 nm		
	8HB	Helix bundles	RNA Pol II-targeting antibodies	8HB: 6 × 6 × 30 nm	Not reported	(118)
	26HB			26HB: 1 × 12 × 90 nm		
Mitochondria	Tetrahedron	DNA tetrahedron	Indirect targeting of mitochondria functions utilizing pharmacological activity of typhaneoside	16.93 ± 3.727 nm	–9.74 ± 3.71 mV	(124)
	Tetrahedron	DNA tetrahedron multiplexes	Triphenylphosphine	10.1 ± 3.4 nm for single tetrahedron 78.8 ± 20.0 nm for aggregates	Not reported	(55)
	Polymeric DNA nanostructures	DNA nanoparticles	Sequence (5′-ACCTTCCTCCGCAGCT-3′)	132.4 ± 11.4 nm	Not reported	(59)
	Oligonucleotide sequences	DNA-zymes with i-motifs	Mitochondrial localization peptide	20–40 bp strands	Not reported	(208)
	Stable DNA duplex	DNA sensor probe coupled with UCNP	Triphenylphosphonium	25 ± 2.8 nm	Not reported	(209)
	DNA nanospheres	DNA nanospheres	Triphenylphosphine	14.9 nm	Not reported	(154)
	DNA probes on polymeric nanoparticles	DNA detection probes conjugated with PLGA-*b*-PEG nanoparticles	Triphenylphosphonium	167.9–246.6 nm	7.82–25.4 mV	(127)
	DNA/Mg^2+^ hybrid nanoflower	DNA/Mg^2+^ hybrid nanoflower containing two aptamers	Cyt C aptamer	∼300 nm	∼ −20 mV	(155)
	DNA-based probes on UCNPs	DNA-based UV activatable probe on a lanthanide-doped UCNP	Triphenylphosphonium	∼100 nm	∼18 mV	(156)
	ATP aptamer on nanoparticles	Redox-responsive ATP aptamer probe on lanthanide-UCNP	Triphenylphosphonium	∼100 nm	∼30 mV	(157), (210)
	DNAzyme integrated on UCNP	UV-activatable DNAzyme sensor probe on UCNP	Triphenylphosphonium	∼100 nm	∼40 mV	(158)
	DNA/Mg^2+^ hybrid nanoflower	DNA/Mg^2+^ hybrid nanoflower modified with cytochrome C aptamer, caging of the photosensitizer [BHQ3-quenched Ce6 (Ce6-Q)], and Cas12a/crRNA complexes	Cyt C aptamer	∼300 nm	∼ −250 mV	(125)
	Y-shaped DNA	Y-shaped DNA	Triphenylphosphine	NA	Not reported	(159)
	photoactivated DNA nanodrug	Incorporation of doxorubicin into C–G base-pair-rich double-stranded DNA and the photosensitizer TMPyP4 into the G-quadruplex	CytC aptamer	221 ± 10 nm	∼ −35 mV	(128)
Lysosomes	61-base pair DNA duplex	61-base pair DNA duplexes modified with Cl^–^ reporter and pH reporter	27-mer duplex	NA	Not reported	(168)
	6HB	Helix bundles	Passive accumulation	6HB: 13.56 ± 3.56 nm	6HB: −21.10 ± 1.11 mV	(58)
	6HB coated with decalysine peptide (K10)			K10:20.10 ± 7.45 nm	K10: −11.10 ± 0.66 mV	
	6HB coated with K10-[aurein 1.2]2 peptide (EE)			EE: 28.52 ± 5.17 nm	EE: −10.90 ± 0.72 mV	
	NIPAM nanoframework	NIPAM, *N*,*N*-methylene diacrylamide (Bis), 3-acrylamidophenylboronic acid and acrylamide-modified dsDNA initiators proton-driven dynamic assembly	Passive accumulation	480 ± 78 nm	Not reported	(56)
	DNA nanocircles	Self-linking of 6HB, decorated with ligands for EGFR and PDL1	IGFIIR aptamer	∼200 nm	Not reported	(134)
Plasma membrane	54 double-helical DNA domains packed on a honeycomb lattice	six double-helical DNA domains that form a hollow tube + barrel-shaped cap that adhered to the membrane composed of 26 cholesterol moieties	Cholesterol moieties	length 42 nm, diameter 2 nm	Not reported	(173)
	DNA nano-octahedron	Enveloped DNA nanostructures in PEGylated lipid bilayer	Artificial lipid membrane	50 nm	Not reported	(211)
	DNA nanopores	Six hexagonally arranged DNA duplexes that are interlinked via hairpins at their termini carrying three cholesterol anchors	Cholesterol moieties	9 nm in height, 5 nm in outer width	Not reported	(174)
	DNA nanopores	Six hexagonally arranged DNA duplexes that are interlinked via hairpins at their termini carrying three cholesterol anchors	Cholesterol moieties	9 × 5 × 5 nm	Not reported	(175)
	DNA nanopores	Six hexagonally arranged DNA duplexes that are interlinked via hairpins at their termini carrying three cholesterol anchors	Cholesterol moieties	length 9.0 ± 1.5 nm; width 5.1 ± 1.1 nm	Not reported	(176)
	6HB	DNA nanobarrels composed of six DNA duplexes which contain three, one, and zero cholesterol lipid anchors	Cholesterol moieties	9 × 5 × 5 nm	Not reported	(177)
Endoplasmic reticulum	DNA tetrahedron	Tetrahedra modified with glucose oxidase	Methyl sulfonamide group and carboxyl group linked together	7 nm	Not reported	(178)
Phagosomes	50 bp DNA duplex	DNA duplexes functionalized with HOCl-sensing dye	Zymosan particles	NA	Not reported	(172)

a Abbreviations: HB, Helix bundles; CMV, Cytomegalovirus; bGH polyA signal, The bovine growth hormone polyadenylation signal; SV40, Simian virus 40; DTS, DNA nuclear targeting sequences; EGFP, Enhanced green fluorescent protein; RNA Pol II, RNA polymerase II; Exo III, Exonuclease III; PLGA-*b*-PEG, Poly(d,l-lactic-*co*-glycolic acid)-*block*-poly(ethylene glycol); Cyt C, Cytochrome C; UV, ultraviolet; UCNP, Upconversion nanoparticle; NIPAM, *N*-isopropylacrylamide; CI-M6PR, also known as IGFIIR, Cation-independent mannose-6-phosphate receptor; EGFR, Epidermal growth factor receptor; PDL1, Programmed death-ligand 1; NA, Not available.

**Figure 2:**
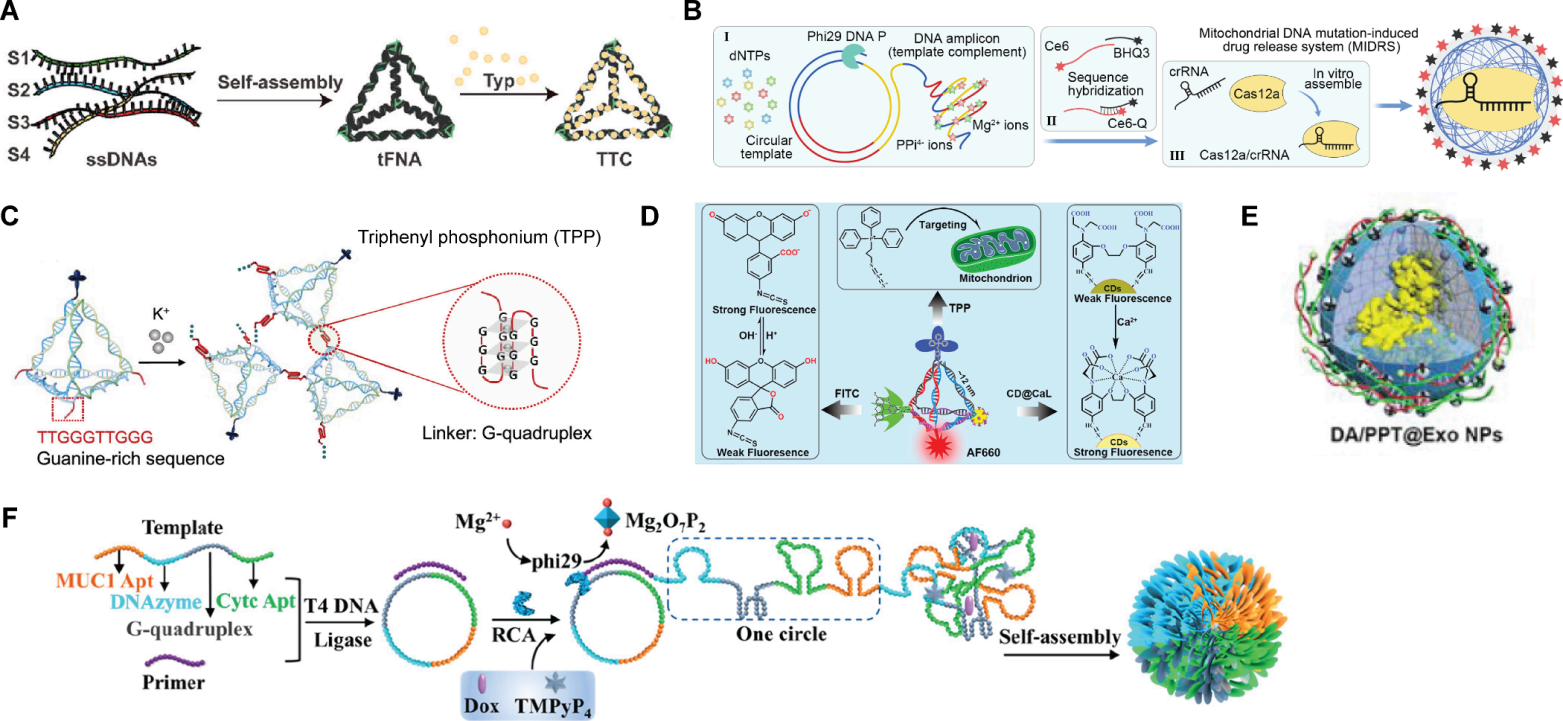
DNs for mitochondrial delivery. (A) Schematic diagram of the production of tetrahedral framework nucleic acid (tFNA) as a vehicle and its combination with typhaneoside (Typ) to develop the tFNA–Typ complex (TTC). Reproduced from ref (124). Copyright 2023 American Chemical Society. (B) Diagram of the design and preparation of a mutation-induced drug release system (MIDRS). Reproduced from ref (125). CC BY-NC 4.0. (C) Schematic illustration of K^+^-mediating aggregation of DNA tetrahedron (denoted as TDN) modified with triphenylphosphine (TPP) for mitochondria targeting. Reproduced from ref (55). Copyright 2022 American Chemical Society. (D) Structure and design of tetrahedron DNA-based nanoprobe. Reproduced from ref (126). Copyright 2018 American Chemical Society. (E) Schematics of DNA detection probes conjugated on Exo III encapsulated polymeric nanoparticles via consecutive adenines (polyA). Reproduced from ref (127). Copyright 2022 American Chemical Society. (F) The synthesis process of MCD@TMPyP4@DOX. A straight-stranded DNA template encodes complementary sequences of MUC1 aptamer, CytC aptamer, DNAzyme, and G-quadruplex with primers in the presence of T4 DNA ligase to form a circular DNA template, followed by RCA reaction with the photosensitizer TMPyP4 and the chemotherapeutic agent DOX to generate the self-assembled DNA nanodrug MUC1 Apt/CytC Apt/DNAzyme@TMPyP4@DOX (MCD@TMPyP4@DOX). Reproduced from ref (128). Copyright 2023 American Chemical Society.

Unlike nucleus-targeting strategies, targeting mitochondria with DNs tends to rely on more conventional approaches (Table 1), like standard modifications by triphenylphosphine or triphenylphosphonium molecules, or cytochrome C aptamers (Table 1 and Figure 2). These established targeting methods have proven effective in directing various types of nanomaterials to mitochondria in previous studies.^129^ Choosing lipophilic cations and mitochondrial targeting peptides is the primary strategy for mitochondrial targeting (Table 1 and Figure 2). The successful targeting of mitochondria relies on membrane potential-driven accumulation and the small size of DNs.^55^ However, potential off-target effects and DN-induced mitochondrial toxicity pose significant challenges for DN-based mitochondrial targeting.^59^ Additionally, overcoming multiple membrane barriers remains a key technical hurdle.^103^ Finally, if targeting the plasma membrane of a cell is the desired effect, functionalizing DNs with cholesterol moieties has been demonstrated to be an effective strategy (Table 1).

Many nanoformulations rely on clathrin-mediated endocytosis, enabling active accumulation in lysosomes without specific functionalization, thanks to the molecular background of the endocytic pathway.^110,130^ Receptor-mediated endocytosis enhances the likelihood that nanoparticles will reach the lysosomes, making ligand modifications essential for effective lysosomal targeting.^110,130,131^ The receptor–ligand interaction can be facilitated by the formation of transport vesicles due to nanoparticle functionalization with specific aptamers targeting tumor cell surface receptors, such as transferrin or antihuman epidermal growth factor receptor-2 monoclonal antibodies.^110,130,131^ These vesicles are subsequently directed through the early endosome, late endosome, and lysosome pathways, leading to the final accumulation of nanoparticles in the lysosomes.^110,130,131^ Lysosomal-targeting molecules, such as alkylated piperidine fragments, can also be used to promote lysosomal accumulation, sometimes self-assembling into anticancer prodrug molecules.^132^ In addition to surface modifications, other physicochemical properties of nanoparticles further influence the efficiency of lysosomal accumulation.^110,130,131^

**Figure 3:**
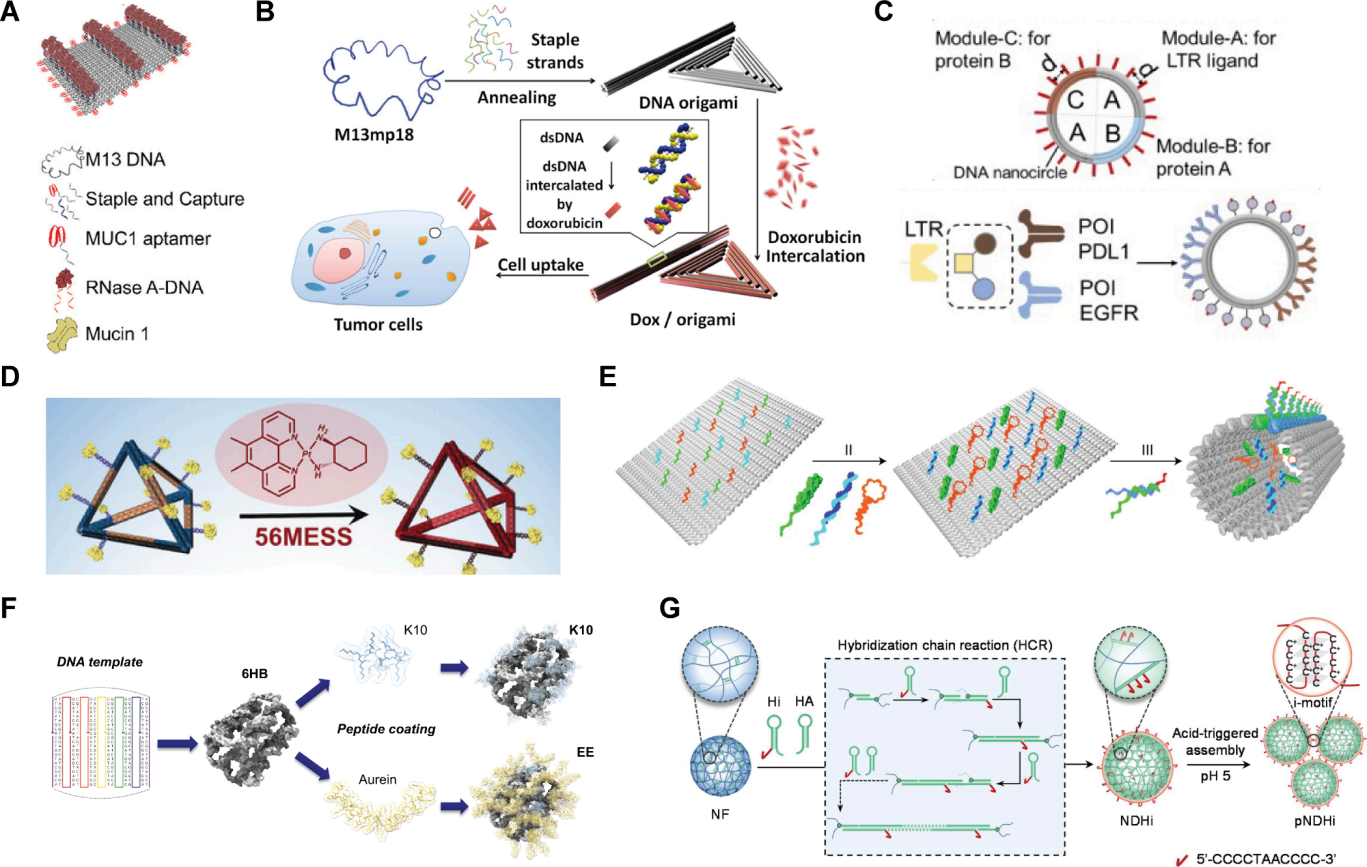
DNs for lysosomal delivery. (A) Schematic illustration of the self-assembly of functionalized DNA nanostructure for lysosome delivery. Tumor targeting strands (red) containing MUC1 aptamer sequences are positioned at four edges of the rectangular origami. Reproduced from ref (133). Copyright 2019 American Chemical Society. (B) DNA origami and doxorubicin origami delivery system assembly. The long single-strand M13mp18 genomic DNA scaffold strand (blue) is folded into the triangle and tube structures through the hybridization of rationally designed staple strands. Watson–Crick base pairs in the double helices serve as docking sites for doxorubicin intercalation. Reproduced from ref (68). Copyright 2012 American Chemical Society. (C) Design principles of intelligent modular DNA lysosome targeting chimeras for combined targeting and immunity therapy of hepatocellular carcinoma (HCC). Reproduced from ref (134). Copyright 2024 American Chemical Society. (D) Schematic illustration of a nanobody-conjugated DNA nanoplatform for targeted platinum drug delivery. Reproduced with permission from ref (135). Copyright 2019 Wiley-VCH. (E) Schematic illustration of the construction of the tumor antigen peptide/CpG loop/dsRNA-*co*-loaded robotic nanostructure by DNA origami. (II) Tumor antigen peptide (OVA peptide or other antigens), CpG loop, and dsRNA are loaded onto the surface of the DNA sheet structure by hybridization with capture sequences (blue, green, and orange) that extend from the surface of the rectangular DNA template. (III) Addition of the locking strands leads to the formation of an antigen/adjuvant-*co*-loaded DNA nanodevice vaccine with a tube shape. Reproduced from ref (136). Copyright 2020 the authors of ref (136), under exclusive license to Springer Nature. (F) Design of peptide functionalized DNA nanostructures for control over lysosomal activity in cells. Reproduced with permission from ref (58). Copyright 2024 Elsevier B.V. (G) Preparation of NDHi and proton-driven assembly of NDHi. Reproduced with permission from ref (56). Copyright 2022 Wiley-VCH.

Lysosomal targeting of DNs employs both strategies: accumulation in lysosomes through the endocytic pathway, and targeted delivery using aptamers or lysosomal-targeting molecules (Table 1 and Figure 3). In summary, the primary strategies for lysosomal targeting involve the use of pH-responsive elements and endocytosis-promoting ligands (Table 1 and Figure 3). For successful lysosomal targeting, key factors include the stability of DNs in the acidic environment of the lysosome and efficient cellular uptake.^102^ Thus, lysosomal degradation and limited escape to cytosol are found to be main challenges for lysosomal targeting.^137^ The other technical hurdle is to achieve a balance between targeted retention within the lysosome and controlled escape into the cytosol.^122^

To the best of our knowledge, functional targeting of other organelles, such as ER, Golgi apparatus, and phagosomes, has been rarely explored (Table 1). These targeting strategies typically involve the use of specific molecular motifs designed to direct nanoparticles to these organelles, e.g. ER-retention signals, and lipid bilayer interactions (Table 1). The success of targeting largely depends on the ability of DNs to escape the endosome, and the precision of ER and Golgi binding.^138^

Targeting the ER, Golgi apparatus, and phagosomes with DNs presents significant challenges due to their complex membrane dynamics, organelle-specific functions, and the need for precise delivery mechanisms.^48,100,101^ The membranes of both the ER and Golgi apparatus are composed of distinct lipid bilayers,^139^ which can hinder the entry of large DNs. For example, the Golgi apparatus is rich in phosphoinositides, which are crucial for membrane trafficking and organelle identity,^139^ complicating the efficient delivery of DNs.

While some studies have explored the use of cell-penetrating peptides and aptamers to enhance delivery, these strategies still face challenges in ensuring that the nanostructures reach the ER or Golgi without being diverted to other cellular compartments.^31,106−108,140^ Additionally, the complex trafficking pathways within the cell, as well as the potential for DNs to induce ER stress, add further hurdles to successful targeting.^138^ Moreover, the structural complexity of the Golgi apparatus presents a technical challenge for precise targeting within its multiple subcompartments.^106^

One other possible explanation for the limited research on targeting the ER, Golgi, and phagosomes with DNs is that this area of research is still in its early stages and requires further development. As this field matures, more effective strategies may emerge to overcome these challenges.

**Figure 4:**
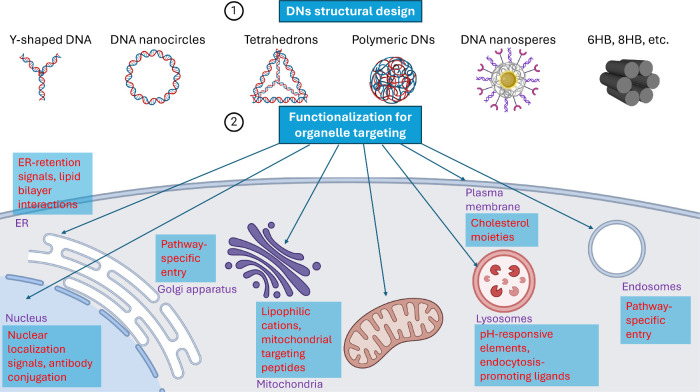
Schematic overview of organelle-targeting strategies using DNs. The synthesis process for DNs designed for specific organelle targeting begins with the careful design of the DNA nanostructure, including considerations of its size, shape, and overall architecture (1). This is followed by functionalization with specific moieties or ligands that enable precise targeting of the desired organelle (2). Created with BioRender.

In summary, we can conclude that the current structural design of DNs (ranging from tetrahedra and origami to nanocages and nanodevices) along with their functionalization, enables effective targeting of distinct cellular organelles, such as the nucleus, mitochondria, lysosomes, plasma membrane, ER, Golgi apparatus, and endosomes (Figure 4), while targeting strategies for ER and Golgi apparatus still await breakthrough improvements.

## 3 Mechanisms of Organelle Interference by DNA Nanostructures

Strategies designed to enhance the efficacy of therapeutic agents through organelle-targeting have been actively researched for nearly 70 years.^111^ By focusing on specific subcellular organelles, these strategies elicit unique biological effects, enabling precise regulation of cellular processes and providing deeper insights into organelle functions.^48,111^ The foundational principles of organelle-targeting strategies include analyzing organelle spatial structures, exploring organelle-mediated death signaling pathways, and developing targeted therapeutic agents.^48,111^

While organelle-targeting therapies have achieved notable successes in treating certain diseases, such as Gaucher and Fabry diseases, significant challenges remain.^48,111^ For example, achieving cellular and tissue-level specificity for organelle-targeting drugs is a major hurdle, compounded by incomplete understandings of the molecular pathogenesis of some diseases, the heterogeneity observed in treatment responses,^48,110,111^ and variability in individual responses to identical treatments.^48,110,111^ Moreover, the efficacy and safety of organelle-targeted treatments require extensive validation.^48,110,111^ Although organelle-targeting nanomedicines improve drug performance, they inherit limitations similar to those of small-molecule drugs, including systemic toxicity and instability.^110^ Finally, complex nanoformulations often exhibit low clinical translation efficiency due to their sensitivity to intracellular environmental changes and structural instability, which can lead to systemic toxicity.^8,110^

DNA nanotechnology offers a promising solution to these challenges by using the precise assembly of DNA strands through complementary base pairing.^24,26−30^ This approach enables the development of diverse encoding and selection strategies, allowing the assembly of molecular building blocks and facilitating targeted interactions.^24,26−30^ Advanced tools, such as DNA walkers and DNA-templated polymer synthesis, exploit DNA’s self-assembly properties to design complex nanostructures with controlled properties (e.g., size, shape, surface functionalization, integration of multiple functional components).^24,26−30,51,63^ These innovations pave the way for rational targeting and modulation of organelle functions, offering precise control over cellular functionality.

Recent advancements propose the construction of exogenous DNA nanostructure aggregates for rational interventions to regulate cell fate, thereby shedding light on molecular mechanisms of disease and providing precise therapeutic solutions at the subcellular level.^107^ This emerging concept, termed “biointerference”, demonstrates the potential of exogenous DNA aggregates for modulating both cell behavior and organelle functions.^107^ For instance, DNA aggregates have been used to interfere with cytoskeletal dynamics, redox homeostasis, mitochondrial functions, and lysosomal processes.^55,56,102,141^ We suggest that beyond DNA aggregates, other DNA nanostructures can be similarly employed to achieve rational control of organelle functions.

In the previous section, we reviewed the current state-of-the-art knowledge on DN design and functionalization for specific organelle targeting. We summarized the strategies that enable organelle targeting. In this section, we will discuss the cellular functions that can be modulated in a controlled manner using DNs. This section highlights critical studies on the development of organelle-targeted DNA nanomaterials and their role in the rational control of cellular functions. To provide an organized overview, we summarize these key studies in Table 2, focusing on their impact on biological functions and potential biomedical applications. Similar to Table 1, this summary is categorized based on the specific organelles targeted.

**Table 2:** Modulation of Cell Functions by Organelle Targeted DNs^a^

organelle	DN format	studied biological system	modulated biological function	potential application	ref
Nucleus	18HB	HEK293T, K562 and primary human T-cells	Delivery of DNs to the cell nuclei and precise genome editing via CRISPR-Cas9	Biosensing in cell nuclei	(115)
	20HB	HEK293T cells	DNA origami enhances gene expression through nuclear import	Active nuclear import of cargo, gene expression modulation	(116)
	20HB, 12HB, 32HB	HEK293T cells	Enhanced gene expression efficiency	Gene delivery applications	(117)
	8HB, 26HB	U2OS cells	Conjugation with RNA Pol II-specific antibodies significantly improved nuclear targeting, intranuclear trafficking	Delivery of nanodevices into live-cell nuclei, biosensing	(118)
Mitochondria	Tetrahedron	HK-2 cells, C57BL/6 mice with acute kidney injury	Increased antiapoptotic and antioxidative effect, and promoted mitochondria and kidney function restoration	Therapeutic potential for acute kidney injury treatment	(124)
	Tetrahedron	smooth muscle cells, MCF-7 cells	Inhibition of the aerobic respiration function of mitochondria and the associated glycolysis process, reduced the production of intracellular ATP, inhibition of lamellipodium formation and cell migration	Potential for suppressing cancer cell migration	(55)
	Polymeric DNA nanostructures	BEAS-2B and A549 cells	Increasing ROS production, decreasing mitochondrial membrane potential, elevation of Ca^2+^ level, decreased ATP level, and inhibition of cell migration	Potential for suppressing cancer cell migration	(59)
	Oligonucleotide sequences	PANC-1, HeLa, HEK293, and T-cells	Enhanced T-cell/cancer cell interactions, increased mitochondrial aggregation and ROS productions, triggering cancer apoptosis	Potential for combination therapy for cancer	(208)
	Stable DNA duplex	MCF-7 and L02 cells, tumor-bearing BALB/c nude mice	Targeted localization of mitochondria, allowing for spatially confined imaging of two cancer-related mitochondrial miRNAs with enhanced detection accuracy	Specific imaging of mitochondrial miRNAs, potential for cancer diagnostics	(209)
	DNA nanospheres	MCF-7, L02, and MDA-MB-231 cells	Imaging of cancer-related mitochondrial miRNAs, detecting cell apoptosis	Specific imaging of mitochondrial miRNAs, potential for cancer diagnostics and screening of anticancer drugs	(154)
	DNA probes on polymeric nanoparticles	HeLa, MCF-7, and A549 cells	Imaging of cancer-related mitochondrial miRNAs, tracking mitochondrial miRNA’s function	Sensitive mitochondrial miRNAs imaging in living cells, potential for cancer diagnostics	(127)
	DNA/Mg^2+^ hybrid nanoflower	HepG2, MDA-MB-231, MCF-7, and A549 cells, tumor-bearing BALB/c nude mice	Efficient imaging of mtDNA mutations in live cells	Potential for cancer diagnostics and metastases detection	(155)
	DNA-based probes on UCNPs	Huh7, HeLa and Huh7 cells, tumor-bearing BABL/c nude mice	Imaging of mitochondria APE1 translocation in response to oxidative stress in live cells	Potential for cancer diagnostics and metastases detection	(156)
	ATP aptamer on nanoparticles	MCF-7 and HEK293T cells, MCF-7 tumor-bearing BABL/c nude mice	Imaging ATP and glutathione in mitochondria	Potential for cancer diagnostics and metastases detection	(157), (210)
	DNAzyme integrated on UCNP	U87 cells	Mitochondria-specific imaging of Zn^2+^ in living cells	Potential for monitoring of ischemia insult and injury	(158)
	DNA/Mg^2+^ hybrid nanoflower	B16, RAW 264.7, HCT116, MUVEC, and MCF-7 cells, B16 tumor-bearing nude mice	Gene silencing and inhibition of mitochondrial respiratory functions	Potential for immunotherapy of cancer	(125)
	Y-shaped DNA	MCF-7, BEAS-2B cells, MCF-7 tumor-bearing BABL/c nude mice	Mitochondria dysfunction, resulting in ATP deficit and hampering cell migration and proliferation, activation of apoptosis of cancer cells and suppression of tumor growth	Potential for anticancer therapy	(159)
	photoactivated DNA nanodrug	MCF-7 and MCF-7/ADR cells, MCF-7/ADR tumor-bearing BABL/c nude mice	Silencing drug-resistant genes, generation ROS, damaging mitochondria, killing cancer cells	Potential for anticancer therapy	(128)
Lysosomes	61-base pair DNA duplex	Primary human skin fibroblasts, cells derived from patients with Niemann–Pick disease, BHK-21, J774A.1, and T47D cells	Quantitative detection of pH and chloride simultaneously in the same lysosome while retaining single-lysosome information in live cells	Potential for lysosomal diseases diagnostics, monitoring disease progression or evaluating therapeutic efficacy	(168)
	6HB	PLC/PRF/5, Huh7, HepG2, and HEK293T cells	Modulation of lysosomal functions, triggering STING activation, lysosomal rupture and mitochondrial damage, resulting in significant cytotoxicity	Potential for the development of combinatorial anticancer platforms, efficient systems for endolysosomal escape, and nanoplatforms modulating lysosomal pH	(58)
	NIPAM nanoframework	A549 and BEAS-2B cells	Lysosomal acidity reduction and hydrolase activity attenuation, thus hindering the degradation of nucleic acid drugs in the lysosome and improving gene silencing effects	Potential for targeted intracellular delivery of drugs	(56)
	DNA nanocircles	HepG2 and MDA-MB-231 cells, HepG2 tumor-bearing BABL/c nude mice	Delivery of EGFR and PDL1 ligands, degradation of EGFR and PDL1, triggering tumor necrosis and inhibition of tumor growth	Potential for anticancer therapy	(134)
Plasma membrane	54 double-helical DNA domains packed on a honeycomb lattice	Artificial small unilamellar vesicles and giant unilamellar vesicles membrane models	DNs inserted into a lipid bilayer membrane, acted as a membrane channel	Potential for antimicrobial agents and interference with cellular homeostasis	(173)
	DNA nano-octahedron	Primary splenocytes and C57Bl/6 mice	Plasma membrane interaction and Immune activation	Potential for immunotherapy	(211)
	DNA nanopores	Polymer-supported membranes	Membrane insertion and artificial pore formation	Potential for controlled transport of various molecules through membrane	(174)
	DNA nanopores	Small unilamellar vesicles	Membrane insertion and artificial pore formation	Potential for controlled transport of various molecules through membrane	(175)
	DNA nanopores	Small unilamellar vesicles	Membrane insertion and artificial mechanically gated ion channel	Potential for controlled transport of various molecules through membrane	(176)
	6HB	Erythrocytes, peripheral blood mononuclear cells and granulocytes	Membrane targeting and suppressing the immune response to pro-inflammatory endotoxin lipopolysaccharide	Potential for vaccine development, immunomodulatory therapy	(177)
Endoplasmic reticulum	DNA tetrahedron	4T1 and BF cells, 4T1 tumor-bearing BABL/c mice, BF tumor-bearing C57 mice	Induction of ER stress, triggering immunogenic cell death of tumor cells, promotion of dendritic cell maturation, stimulation of T cell proliferation and infiltration	Potential for cancer immunotherapy	(178)
Phagosomes	50 bp DNA duplex	SIM-A9, J774A.1 and RAW 264.7 cells, monocytes, neutrophils, bone marrow derived macrophages, adipose tissue macrophages, peritoneal macrophages	Mapping phagosomal production of a specific ROS, HOCl, as a function of phagosome maturation	Potential for tracking immune activation	(172)

a Abbreviations: HB, Helix bundles; UCNP, Upconversion nanoparticle; RNA Pol II, RNA polymerase II; ROS, Reactive oxygen species; miRNAs, Micro ribonucleic acids; mtDNA, Mitochondrial DNA; APE1, Apurinic/apyrimidinic endonuclease 1; STING, Stimulator of interferon genes; EGFR, Epidermal growth factor receptor; PDL1, Programmed death-ligand 1; NIPAM, *N*-isopropylacrylamide; ER stress, Endoplasmic reticulum stress.

### 3.1 DNA Nanostructures for Interfering with the Nucleus

The nuclear envelope represents a significant barrier to efficient gene delivery due to its selective permeability, which prevents the free passage of macromolecules such as exogenous genetic material.^142^ This obstacle remains one of the major challenges in achieving effective nuclear import for gene therapy.^142,143^ It is widely accepted that the primary route for exogenous DNA to access the nucleus involves passive entry during mitosis, a process during which the nuclear envelope temporarily disassembles, allowing cytoplasmic contents to mix with the nuclear compartment. However, this mechanism is inherently inefficient, resulting in only a small fraction of the DNA successfully reaching the nuclear interior.^142−144^

DNs have been widely investigated in the context of delivery and cellular uptake.^145−147^ However, the targeted delivery of these nanostructures specifically to the nucleus has only recently become a focus of research (Table 2).^115−117,148^ Utilization of DNs primarily as vehicles to deliver genetic material has already shown great potential in gene delivery, since ensuring the precise transport of genetic material to the nuclear compartment is critical for therapeutic efficacy (Table 2).^115−117,148^ The flexibility and programmability of DN functionalization—combined with the fact that they can be inherently comprised of the genetic material to be delivered—enables various possibilities to target the nucleus and manipulate cell functionality (Table 2). For example, DNA constructs encoding both an intact human gene and a fluorescent protein gene have been demonstrated to serve as highly effective, compact templates for precise gene integration through CRISPR-mediated homology-directed repair.^115^ CRISPR-Cas9 homology-directed repair (HDR) has been employed as a strategy for the nuclear delivery of DNs. In this approach, 18-helix nanostructures, which carry CRISPR-Cas9 binding sites at their ends, were utilized.^115^ These DNs were successfully applied in HDR-mediated genome editing, demonstrating their ability to compact DNA and facilitate precise localization within the nucleus.^115^ This innovative strategy not only enhances DNA delivery but also has the potential to expand the range of CRISPR applications,^115^ offering new options for targeted genome modifications.

DNs have shown significant potential in enhancing gene expression efficiency by incorporating and optimizing various functional sequences and structural elements.^117^ Recent studies have demonstrated that DNs can unfold in the intracellular environment, with this unfolding potentially exposing the single-stranded DNA (ssDNA) scaffold, thereby allowing gene expression to occur from the bare scaffold strand.^117^ The efficiency of this gene expression can be further improved through targeted design strategies. Among the most promising strategies are virus-inspired inverted-terminal repeat-like (ITR) hairpin motifs, which are strategically positioned upstream or flanking the expression cassette.^117^ These motifs mimic natural viral elements that enhance both gene expression and stability, contributing to the overall effectiveness of the delivered genetic material. This ability to finely tune functional sequences and structural designs underscores the versatility of DNA nanostructures for advanced gene delivery applications (Table 2).^117^ Moreover, the study demonstrated the successful codelivery of genes in controlled stoichiometric ratios.^117^ To evaluate the functionality of DNs in gene delivery and investigate their intracellular behavior, the DNs were modified with reporter genes (e.g., mCherry, EGFP).^117^ These modifications allowed for the tracking and quantification of the gene delivery process. However, the authors propose that DNs can be further customized for specific gene delivery applications by replacing the fluorescent reporter genes with sequences that encode custom proteins.^117^

**Figure 5:**
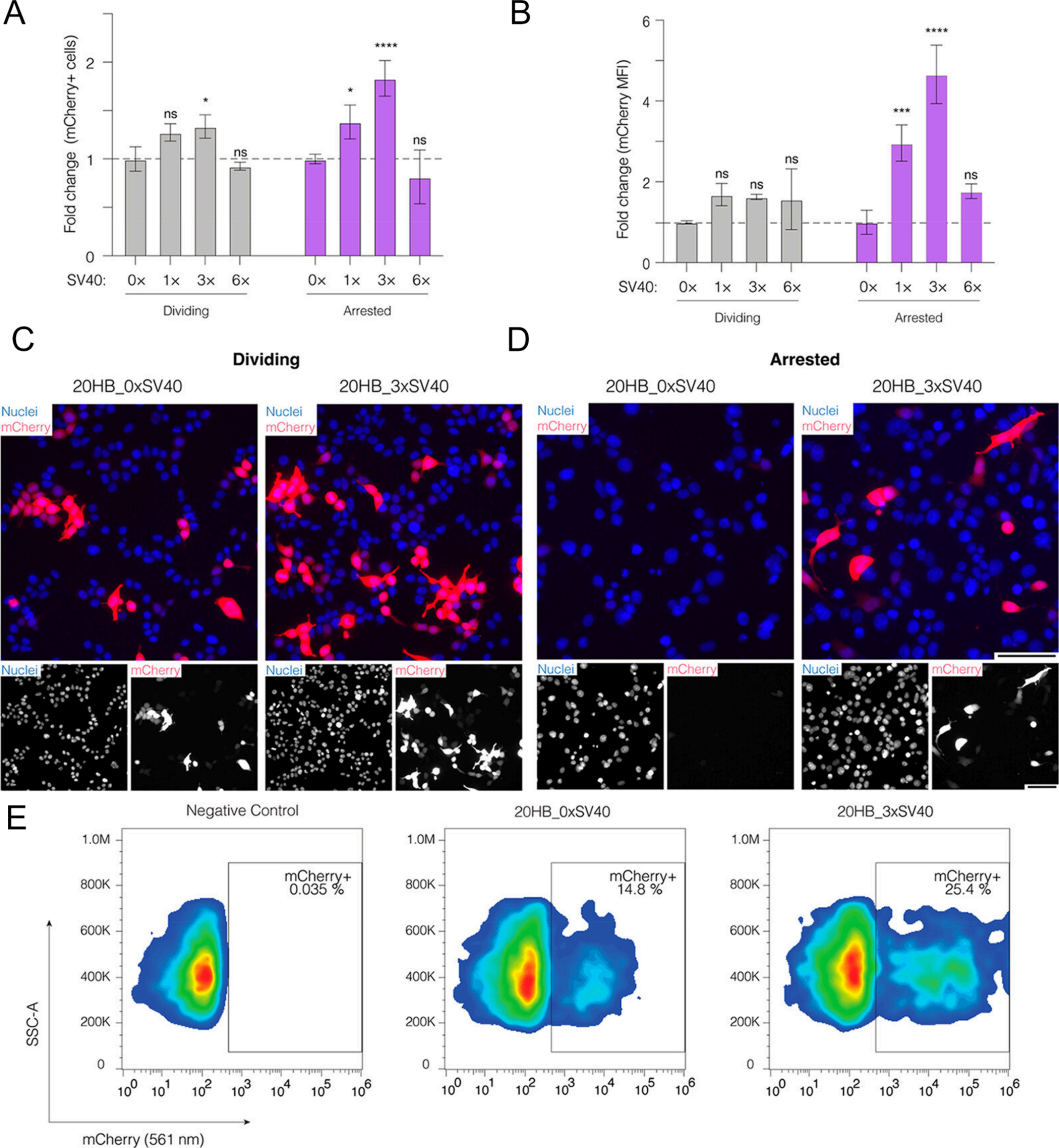
Presence of SV40 DTS sequences in DNA origami enhances gene expression through nuclear import. (a) Fold change of the percentage of mCherry + cells and (b) mean fluorescence intensity (MFI) of mCherry in dividing and arrested cells after electroporation with 20HB variants. Both the percentage of mCherry positive cells and MFI are shown as fold change compared to the value of the control 20HB_0×SV40 in dividing and arrested cells, respectively. Data collected in (a) and (b) were quantified using flow cytometry and are presented as mean ± standard deviation (s.d.) for *n* = 3 biologically independent experiments. Statistical analysis was performed using two-way ANOVA with Dunnett’s multiple comparisons (*p ≤ 0.05, ***p ≤ 0.001, ****p ≤ 0.0001, ns *p* > 0.05). (c, d) Representative epifluorescence microscopy images after electroporation of dividing cells (c) and arrested cells (d) for the control 20HB_0×SV40 and 20HB_3×SV40. Images were taken 24 h after electroporation and are representative of *n* = 3 biological replicates (similar results were observed each time). In overlay, the mCherry signal is shown in red, and nuclei are shown in blue. Scale bar is 100 μm. (e) Representative flow cytometry gates demonstrating mCherry expression (mCherry 561 nm, *x* axis) against side-scatter area (SSC-A, *y* axis) in chemically arrested HEK293T cells. Reproduced from ref (116). CC BY 4.0.

DNs have also been identified as highly effective platforms that integrate gene-encoding sequences, a reporter gene (mCherry), and multiple Simian virus 40 (SV40)-derived DNA nuclear targeting sequences (DTS) within a single construct.^116^ This combination facilitates enhanced active nuclear import of the nanostructures, which in turn significantly improves the efficiency of the desired gene expression (Figure 5).^116^ One advantage to DNs (generated using the origami approach) is that the gene to be delivered can itself be the “scaffold” that is folded into the desired nanoscale shape, rather than needing to package the gene into a separate nanocarrier. As such, this approach can maximize the fraction of the nanoparticle that is the functional element. To assess the efficacy of gene delivery, the authors employed fluorescent protein-encoding reporter genes, such as mCherry. The study found that the inclusion of SV40-derived DTS sequences significantly enhanced gene delivery, particularly in arrested cells, where the percentage of mCherry + cells increased (Figure 5). Overall, the findings demonstrate that intracellular nuclear import of DNs can be effectively induced and increased by displaying virus-derived DTS sequences on the surface of DN particles.

Alternatively, DNs coupled with an antibody targeting Pol II have been shown to be actively transported into the nucleus.^118^ Once delivered, these nanostructures demonstrated stability within cells for up to 24 h, including sustained presence inside the nucleus.^118^ These results highlight the expanded application potential of DNs beyond their use in gene delivery and manipulation. Furthermore, the authors proposed DNs as suitable probes for biophysical measurements, including the study of chromatin rearrangement and nuclear organization.^118^

Recent studies have demonstrated the enhanced delivery of anticancer drugs to the nucleus, which may significantly improve therapeutic efficacy.^104^ In one such study, folate-modified DNA tetrahedra loaded with mitoxantrone were used to specifically target leukemic cells.^104^ The results showed that these nanostructures could efficiently cross the nuclear membrane, trigger apoptosis, and significantly enhance the therapeutic effect of mitoxantrone in both *in vitro* models and leukemia-bearing mice.^104^ This approach highlights the potential of DNA nanostructures as a targeted delivery system for improving the treatment of leukemia and other cancers.

In summary, targeting the nucleus with DNs holds significant potential for applications in gene therapy and drug delivery. Specifically, the incorporation of NLS and antibody conjugation has been shown to enhance the nuclear delivery of DNs, improving their efficiency and precision.^122^ Nuclear-targeted DNs have demonstrated the ability to modulate gene expression and effectively deliver functional nucleic acids, paving the way for advanced therapeutic strategies.^149^ Moreover, optimizing DN design could further enhance the nuclear delivery of anticancer drugs, thereby increasing their therapeutic efficacy.^104^ Additionally, some advanced DN architectures have facilitated CRISPR-mediated genomic integration,^115^ expanding the potential of DNA nanostructures in gene editing applications and offering new avenues for precision medicine.

### 3.2 DNA Nanostructures for Interfering with Mitochondria

Mitochondria play a crucial role in energy production, calcium metabolism, reactive oxygen species (ROS) generation, and tuning the immune response, making them key regulators of cell death.^150−152^ Furthermore, mitochondria have become prominent targets for anticancer drugs.^150−152^ Therapeutic agents such as lonidamine, amlodipine, ceramide, and natural compounds like resveratrol, berberine, and betulinic acid induce cancer cell death by activating apoptotic proteins (Bax and Bak), and releasing cytochrome *c*.^110,150−152^ Mitochondria in cancer cells are more vulnerable than in normal tissues,^152,153^ providing opportunities to deliver radiosensitizers, photosensitizers, and theranostics to stimulate ROS production and oxidative stress.^110,150−152^ These strategies disrupt mitochondrial permeability, impair energy supply, and drive cancer cell death.^110,150−152^ Consequently, mitochondria-targeting approaches are being actively explored to regulate mitochondrial function and influence cellular fate efficiently.

Similar to their interactions with the nucleus, the structural plasticity and versatility of DNs provide numerous opportunities for rational modulation of biological functions or control of mitochondrial functionality. These range from serving as probes or sensors for mitochondria-specific functions to acting as active therapeutic agents capable of either restoring or disrupting mitochondrial functions on demand (Table 2). For example, several studies have highlighted the potential of DNs as imaging probes for detecting mitochondrial miRNA and/or DNA and monitoring mitochondrial functions (Table 2). These applications demonstrate significant promise in cancer diagnostics, including the detection of metastasis and the screening of anticancer drugs.^127,154,155^ By enabling precise and real-time monitoring of mitochondrial activity and associated molecular changes, DNA nanostructures could provide valuable insights into cancer progression and the efficacy of therapeutic interventions.^127,154,155^ Additionally, DNA nanotechnology has expanded to the development of probes for detecting human apurinic/apyrimidinic endonuclease 1 (APE1), ATP, and glutathione within mitochondria.^156,157^ An innovative DNAzyme-based biosensor technology was recently developed to monitor mitochondrial Zn^2^^+^ levels during drug treatment in a model of ischemic insult.^158^ This technology opens new avenues for applying DNA-based nanomaterials to investigate the pathophysiological roles of metal ions at the subcellular level.^158^

A variety of DNA-based nanomaterials, both standalone and hybrid, have recently been developed with the ability to target mitochondria and strategically modulate key mitochondrial functions. These advancements have led to two primary therapeutic outcomes: either disrupting mitochondrial function to effectively induce cancer cell death or restoring damaged mitochondrial functions to alleviate pathological conditions (Table 2).

For instance, tetrahedral DNA nanostructures and polymeric DNA nanomaterials have been successfully utilized to inhibit the aerobic respiration function of mitochondria, along with the associated glycolysis process in cancer cells.^55,59^ To achieve targeted delivery to the mitochondria, tetrahedral DNs were modified with the mitochondrial targeting compound triphenylphosphine.^55^ These modified tetrahedral DNs induced a reduction in mitochondrial membrane potential,^55^ leading to elevated ROS levels, which in turn caused calcium overload.^55^ As a result, mitochondrial respiratory activity was significantly reduced.^55^ This inhibition results in a significant reduction in intracellular ATP production, thereby impairing the formation of lamellipodia and restricting cell migration.^55,59^ Such effects demonstrate the potential of these nanomaterials to suppress cancer cell migration and reduce the metastatic potential of cancer cells, making them a promising tool for anticancer therapies.

DNs have also emerged as an excellent vehicle for introducing genetic manipulations specifically within mitochondria. For example, DNA/Mg^2^^+^ hybrid nanoflowers engineered to target mitochondria have demonstrated remarkable efficiency in identifying single nucleotide variants (SNVs) in mitochondrial DNA (mtDNA), as well as in splicing mtDNA, inducing gene silencing, and hampering mitochondrial respiration.^125^ Additionally, these nanoflowers facilitate the generation of cytotoxic ROS, which further injured mitochondria and triggered the release of mtDNA into the cytoplasm.^125^ This release activates the cyclic GMP-AMP synthase-stimulator of interferon genes (cGAS-STING) pathway, resulting in the specific killing of tumor cells. Simultaneously, this mechanism promotes dendritic cell maturation and facilitates the infiltration of cytotoxic T lymphocytes into the tumor microenvironment.^125^ More specifically, these complex DNs were functionalized with a cytochrome C aptamer for targeted delivery to the mitochondria.^125^ In addition, the DNs carried the photosensitizer [BHQ3-quenched Ce6 (Ce6-Q)] and Cas12a/crRNA complexes, which are capable of recognizing tumor cell-specific SNVs in mtDNA.^125^ Once delivered to the mitochondria, the DNs cointroduced the Cas12a/crRNA complex, Ce6-Q, and Mg^2^^+^ into the mitochondria.^125^ This enabled the activation of Cas12a’s trans-cleavage activity upon recognizing tumor cell-specific SNVs in the mtDNA.^125^ The photosensitizer Ce6-Q was used to photodynamically activate the tumor cell-specific SNVs in the mtDNA, facilitating precision targeting of mutant mitochondria.^125^ This strategy was effective both *in vitro* and *in vivo*, enabling highly targeted intervention in tumor cells.^125^ Collectively, these nanostructures enable precision tumor therapy with single-cell resolution, showcasing their potential for the rational design of nanomedicines that achieve genotype-specific therapeutic effects.^125^ This innovative approach highlights the significant potential of DNA-based nanomaterials for advancing targeted cancer therapies.

Another distinct approach for forming DNs inside living cells has been proposed, leveraging telomerase-guided intracellular self-assembly.^159^ Y-shaped DNA strands were shown to self-assemble into a DNA network within living cells, which could be specifically targeted to mitochondria.^159^ This mitochondrial targeting induced significant mitochondrial dysfunction, leading to a critical ATP deficit that disrupted cellular energy metabolism.^159^ Y-shaped DNs were modified with triphenylphosphine for targeted delivery to the mitochondria.^159^ Additionally, the structures incorporated a telomerase-responsive linker DNA (T-L-DNA), which contained L-DNA and a telomerase primer for specific telomerase recognition.^159^ This design enabled the release of L-DNA from the T-L-DNA via a telomerase-mediated strand displacement reaction, leading to the formation of a DNA network.^159^ Importantly, this DNA network could only form in cancer cells, as normal cells lack telomerase activity.^159^ Once formed, the DNA network encapsulated the mitochondria, blocking the exchange of substances within the cell.^159^ This resulted in the inhibition of both oxidative phosphorylation and glycolysis, effectively disrupting cellular metabolism in cancer cells.^159^ Consequently, cell migration and proliferation were hindered, triggering apoptosis in cancer cells. Furthermore, this mechanism effectively suppressed tumor growth, showcasing the potential of telomerase-guided self-assembly as a novel strategy for intracellular delivery and targeted cancer therapy.^159^ This approach highlights the versatility of DNA nanotechnology in utilizing endogenous cellular factors, such as telomerase, to enable precise therapeutic interventions at the subcellular level.

**Figure 6:**
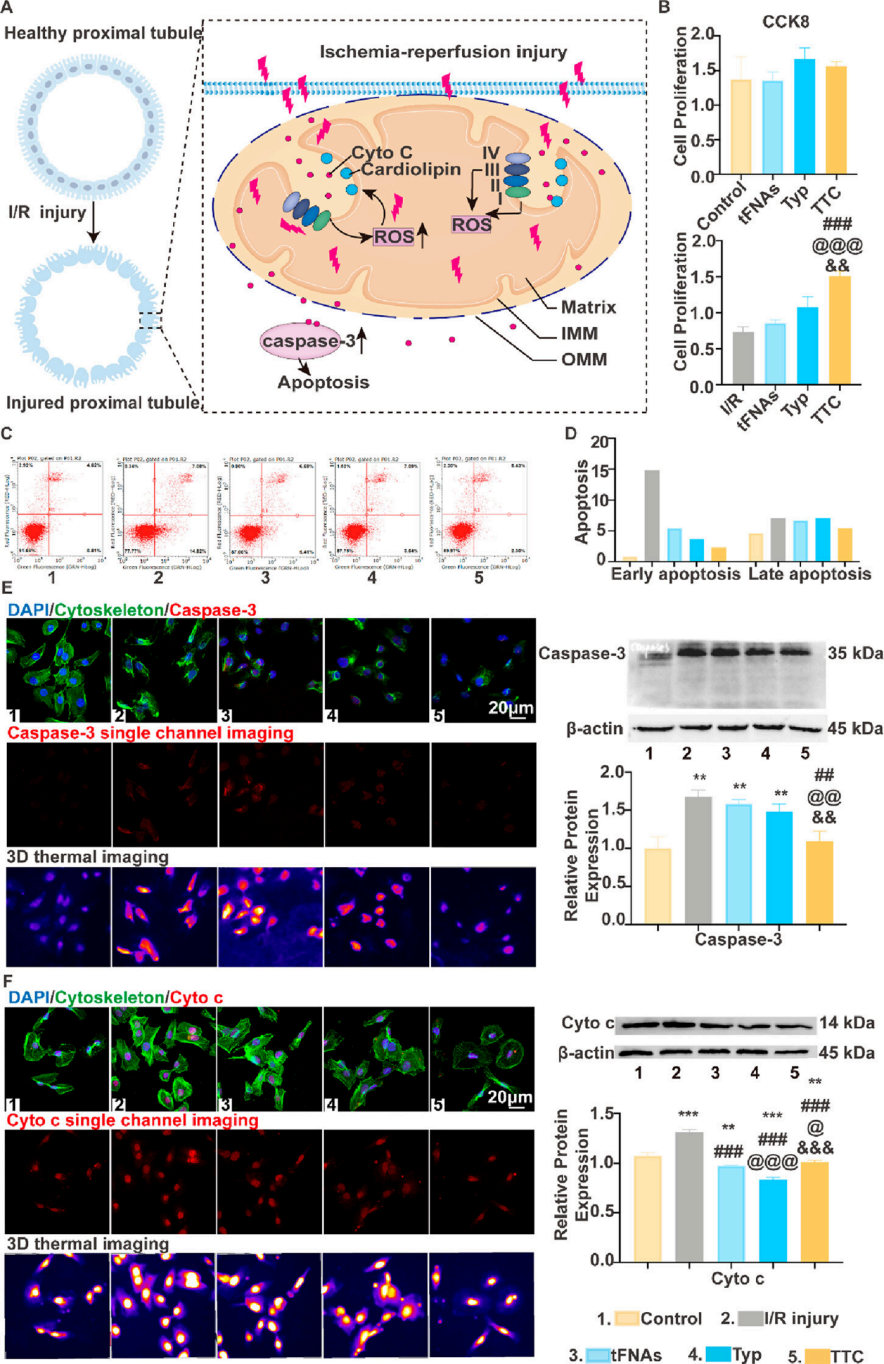
TTC decreased cell apoptosis after I/R Injury. (A) Schematic graph of I/R injured tubular cells. (B) Cell viability of tFNAs, Typ, and TTC on healthy and I/R HK-2 cells analyzed by CCK-8 assay (*n* = 3). (C) Cell apoptosis in five groups: the control group, I/R group, I/R + tFNAs group, I/R + Typ group, and the I/R + TTC group using flow cytometry. (D) Quantification of (C), including early and late apoptosis rate of cells. (E) Expression of Cyto C in HK-2 after tFNAs, Typ, and TTC treatment using confocal microscope and Western blot. Quantification of related proteins calculated by ImageJ (*n* = 3). (F) Expression of Caspase-3 in HK-2 after tFNAs, Typ, and TTC treatment using confocal microscopy and Western blot. Quantification of related proteins calculated by ImageJ (*n* = 3). The statistics are presented as mean ± standard deviation. Statistical analysis: *p < 0.05, **p < 0.01, ***p < 0.001 (Groups: *, control; #, I/R; @, tFNAs; &, Typ). Reproduced from ref (124). Copyright 2023 American Chemical Society.

It is worth noting that DNs have the versatility to not only disrupt mitochondrial function but also to restore it, depending on the therapeutic goal (Table 2). For example, tetrahedral framework nucleic acids functionalized with typhaneoside, a flavonoid with potent antioxidative properties, were successfully targeted to injured mitochondria.^124^ This precise targeting led to enhanced antiapoptotic and antioxidative effects, effectively mitigating mitochondrial damage (Figure 6).^124^ Specifically, typhaneoside-modified DNs were found to reduce both early and late apoptosis rates (Figure 6).^124^ Mechanistically, the DNs inhibited the expression of pro-apoptotic proteins, including cytochrome *c* and caspase-3.^124^ Additionally, the DNs prevented the excessive accumulation of mitochondrial ROS.^124^ Furthermore, treatment with DNs successfully restored mitochondrial dynamics and recovered mitochondrial membrane potential following ischemia-reperfusion injury,^124^ highlighting their potential as a therapeutic strategy to mitigate cellular damage under such conditions. As a result, mitochondrial function was restored, which further contributed to the recovery of kidney function.^124^ This dual action provided significant therapeutic benefits in models of acute kidney injury (AKI).^124^ The ability of tetrahedral frameworks to deliver bioactive molecules specifically to damaged mitochondria not only promotes mitochondrial and cellular repair but also highlights the broader applications of DNA-based nanomaterials in treating various mitochondrial dysfunction-related diseases.

In summary, recent studies highlight mitochondria as key targets for DNs, with promising applications in sensing, drug delivery, and mitochondrial function modulation. DNA nanodevices have enabled spatiotemporally resolved ATP sensing within mitochondria, providing insights into cellular energy dynamics.^138^ Mitochondria-targeted DNs have also demonstrated potential for delivering therapeutic agents directly to these organelles, enhancing treatment precision.^160^ Furthermore, research has shown that DNs can actively modulate mitochondrial function, influencing cellular energy metabolism.^55^ DNA-based probes have facilitated high-resolution imaging and real-time sensing of mitochondrial parameters, such as zinc ion concentrations.^158^ Additionally, advanced DNs have been designed to induce mitochondrial aggregation and fusion, opening new possibilities for manipulating organelle morphology and function.^161^

### 3.3 DNA Nanostructures for Interfering with Endolysosomes

Lysosomes—spherical membranous vesicles containing various hydrolytic enzymes—are essential organelles responsible for the digestion and degradation of cellular waste in eukaryotic cells.^162,163^ However, lysosomes are far more than mere “trash bags”. They play critical roles in maintaining cellular homeostasis, mediating cell apoptosis, regulating nutrient sensing, and modulating immune responses.^162−164^ Dysfunction of lysosomes can have wide-ranging detrimental effects, including disrupted cellular signaling and the induction of apoptosis. In cancer cells, lysosomal properties often undergo significant alterations due to malignant transformation.^165^ These changes include variations in lysosomal volume, composition, and subcellular localization.^165,166^ Increased lysosomal fragility in cancer cells, often associated with elevated levels of sphingomyelin, makes them more prone to lysosomal membrane permeabilization, a process that can trigger cell death.^165−167^ Emerging research has demonstrated that DNs targeting lysosomes represent a promising strategy for manipulating lysosomal function and the microenvironment (Table 2). By modulating these factors, DNs have the potential to influence cellular behaviors and fate, offering innovative therapeutic opportunities for conditions like cancer and lysosome-associated disorders (Table 2).

DNA nanotechnology has introduced systems that can serve as highly effective probes for monitoring lysosomal activity. For example, these systems enable the simultaneous quantitative detection of pH and chloride levels within individual lysosomes while preserving single-lysosome resolution in live cells.^168^ Effective monitoring of lysosomal activity holds significant potential for the diagnosis of lysosomal diseases, tracking disease progression, and evaluating the efficacy of therapeutic interventions.^168^ Additionally, DNs can actively modulate lysosomal activity in a controlled manner, which in turn influences various regulated cellular functions (Table 2). For example, intracellularly self-assembled DNA nanoframeworks have been shown to reduce lysosomal acidity and attenuate hydrolase activity.^56^ This inhibition of lysosomal function prevents the degradation of nucleic acid–based drugs, thereby enhancing the effectiveness of gene silencing.^56^ Specifically, these DNA nanoframeworks demonstrated pH-dependent self-assembly within lysosomes.^56^ The self-assembly process led to the formation of aggregates, which enhanced the retention of the nanoframeworks inside the lysosomal compartment.^56^ Additionally, the protonation of the DNA structures in the acidic lysosomal environment induced interference with lysosomal function, as evidenced by a decrease in acidity and a reduction in the activity of lysosomal hydrolases.^56^

Furthermore, DNA nanocircles functionalized with pH-sensitive DNA motifs, aptamers targeting lysosome-shuttling receptors, and programmed cell death ligand-1 (PD-L1) demonstrated significant therapeutic potential in the treatment of hepatocellular carcinoma (HCC).^134^ These engineered nanostructures effectively facilitated the degradation of epidermal growth factor receptor (EGFR) and PD-L1 in tumor tissue, enabling a dual-targeting strategy that combines a targeted therapeutic effect and immune modulation.^134^

By utilizing lysosome-targeting chimeras, the authors developed intelligent, modular nanodevices designed for tumor-specific degradation of multipathogenic proteins.^134^ These nanodevices integrate dynamic properties and nanoscale control inherent in DNA nanostructures. The devices were meticulously engineered through precise control over the stoichiometry and modularity of pH-responsive lysosome-trafficking receptors, protein degradation promoters, and specific protein ligands, all embedded within circular DNA origami nanostructures. These DNs serve as ideal nanocarriers due to their capacity to integrate structurally complex and carefully prepared ligands, which are essential for facilitating effective, targeted protein degradation.^134^ Notably, the incorporation of pH-sensitive DNA motifs into the IGF-II receptor (IGFIIR) aptamer sequences within the promoter region functions as a molecular switch, triggered by the acidic microenvironment of tumors.^134^ Given the widespread expression of IGFIIR across various tissues, this molecular switch enables selective and precise activation of protein degradation processes. The DNs facilitate accurate localization and delivery of therapeutic agents directly into tumor tissues.^134^ Once in the acidic tumor microenvironment, the degradation switch is activated, enabling multivalent binding and efficient degradation of a range of prespecified target proteins.^134^ The results were highly promising, with pronounced tumor necrosis observed alongside a significant inhibition of tumor growth.^134^ This approach not only enhances the therapeutic efficacy by targeting critical signaling pathways but also boosts immune response by downregulating immune checkpoint proteins like PD-L1, thus fostering a more effective antitumor immune environment.^134^

DNs can be effectively utilized to target organelles closely related to lysosomes, such as endosomes. DNA-based probes have been specifically designed for pH mapping and tracking endocytic pathways, demonstrating their potential for real-time monitoring of cellular processes.^169^ In addition, one study highlighted the use of a DNA nanoswitch incorporating a Förster resonance energy transfer (FRET) donor–acceptor pair to probe the specific recognition of p50/p50 and p50/p65 NF-κB dimers within cells.^169^ This DN-based nanosensor serves as an innovative strategy to track intracellular trafficking and interactions with the NF-κB signaling pathway, offering valuable insights into its dynamic role in cellular processes.^169^ By using DNs as nanosensors, researchers can gain deeper insights into the dynamics of endosomal maturation, protein sorting, and other key aspects of cellular trafficking of furin (a protease playing an important role in activation of number of crucial cellular proteins).^106^ This approach not only enhances our understanding of the cellular internalization processes but also opens new avenues for exploring the regulatory mechanisms involved in protein signaling pathways.^106^

**Figure 7:**
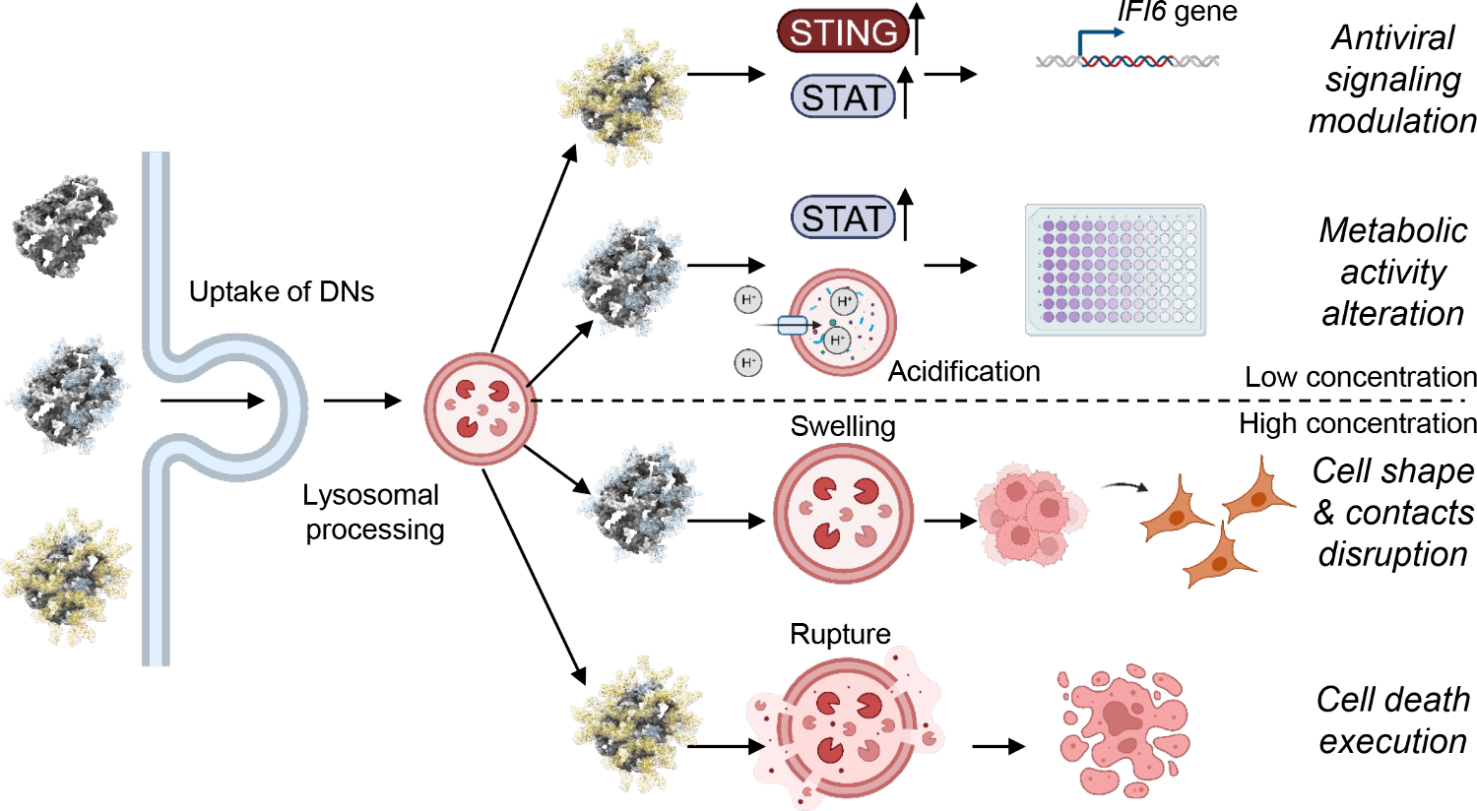
Scheme of the controlled modulation of lysosomal functions by functionalized DNA nanostructures. Created with BioRender. Reproduced with permission from ref (58). Copyright 2024 Elsevier.

Our team has developed a DN-based platform capable of modulating a wide range of cellular functions, with the ability to fine-tune these effects based on the concentration and surface decoration of the nanostructures (Figure 7).^58^ By using structurally different peptides for surface functionalization, we were able to precisely control lysosomal functions, which in turn influenced various cellular processes, including changes in cell morphology, immune signaling modulation, and the regulation of cell death (Figure 7).^58^ At low concentrations, DNs functionalized with decalysine peptide promoted lysosomal acidification, which in turn altered the metabolic activity of vulnerable cells.^58^ This shift in lysosomal pH had profound effects on cellular homeostasis and metabolic pathways. On the other hand, DNs coated with aurein (and endosome-escaping peptide) caused lysosomal alkalization, resulting in activation of the STING pathway, a crucial element in the innate immune response.^58^ At higher concentrations, decalysine peptide-coated DNA nanostructures induced lysosomal swelling, loss of cell–cell contacts, and morphological changes, but did not cause cell death (Figure 7).^58^ These results suggest a nonlethal, yet disruptive, modulation of lysosomal dynamics. In contrast, high concentrations of aurein-coated DNA nanostructures led to lysosomal rupture and mitochondrial damage, ultimately resulting in significant cytotoxicity.^58^

To summarize this section, we can conclude that DNs have demonstrated significant potential in targeting lysosomes, with applications spanning from pH sensing to drug delivery (Table 2). Several studies have leveraged pH-responsive DNs to track the acidification processes occurring in endosomes and lysosomes, providing valuable insights into their dynamic behavior (Table 2).^106^ In addition, DNs have been utilized to effectively deliver therapeutic agents to lysosomes, potentially improving the efficacy of lysosome-targeted therapies.^170^ The precise design of DNs has enabled the investigation of lysosomal heterogeneity and the identification of distinct lysosomal subpopulations, contributing to a deeper understanding of their functional diversity.^171^ Furthermore, the trafficking of DNs through the endolysosomal pathway has provided critical insights into the mechanisms governing cellular uptake, thereby enhancing our knowledge of the cellular internalization process.^122^ Additionally, DNs functionalized with specific peptides offer a versatile approach for modulating lysosomal activity,^58^ presenting opportunities for therapeutic interventions aimed at lysosomal dysfunction.

### 3.4 DNA Nanostructures for Interfering with Other Organelles

Although DNs are primarily investigated as tools for the rational regulation of nuclear, mitochondrial, and lysosomal functions, recent studies highlight their potential to selectively target other organelles, such as the plasma membrane, ER, and phagosomes (Table 2). As observed with other organelles, DNA-based systems can be effectively utilized as sensors to monitor specific cellular processes (Table 2). For example, these systems can be employed to map the production of ROS, such as hypochlorous acid, within phagosomes during their maturation.^172^ This capability demonstrates the potential of DNA-based sensors in tracking immune activation and providing valuable insights into the dynamics of immune responses.^172^

Plasma membrane targeting has been demonstrated using various DN platforms, such as 54 double-helical DNA domains arranged on a honeycomb lattice, DNA nano-octahedra, and DNA nanopores composed of six-helix bundles modified with cholesterol (Table 2). These systems were designed to be inserted into lipid bilayer membranes, facilitating artificial pore formation and acting as membrane channels.^173−176^ The implications of these interactions hold potential for the controlled transport of various molecules, including antimicrobial agents, and for the modulation of cellular homeostasis. Plasma membrane targeting using cholesterol-modified six-helix bundles enabled selective targeting of white blood cells, and modulating their activity by suppressing the immune response to pro-inflammatory endotoxins, such as lipopolysaccharides.^177^

ER targeting was successfully achieved using DNA tetrahedra functionalized to bind specifically to the sulfonamide receptor on cancer cell membranes.^178^ Upon binding, these nanostructures induced glucose depletion and excessive ROS production, which triggered a robust ER stress response.^178^ At the mechanistic level, the DNA nanostructures accumulated within the ER, where they facilitated glucose oxidation by glucose oxidase, leading to the consumption of glucose and the generation of hydrogen peroxide.^178^ This, in turn, caused a significant accumulation of ROS within the ER. Both the depletion of glucose and the generation of ROS within the ER contributed to the activation of a strong ER stress response, further disrupting cellular homeostasis and promoting cell death pathways.^178^ This response led to immunogenic cell death (ICD), exposing tumor-associated antigens and promoting the activation of the immune system.^178^ As a result, dendritic cell maturation was enhanced, and the proliferation and infiltration of T cells was stimulated, advancing the efficacy of cancer immunotherapy.^178^ This strategy highlights the potential of DNA nanostructures not only for targeted organelle-specific delivery but also as powerful tools for inducing therapeutic stress responses in cancer cells by manipulating metabolic pathways and oxidative stress within critical cellular compartments.

## 4 Outlook and Future Directions

With advancements in bionanotechnology, the research into and applications of subcellular-targeting nanoparticles have emerged as a prominent field of study over the past five years.^110,111^ Significant progress in nanotechnology has accelerated the development of various nanoformulations that specifically target subcellular structures.^110^ DNs, in particular, can be engineered as programmable molecular scaffolds that precisely target specific organelles *in vivo* (Table 2). The unique modularity and precise control over stoichiometry that nucleic acids provide enable the creation of multifunctional systems, making DNs ideal for precision medicine.^51,63,119−121^

These systems can integrate diagnostics and personalized therapies, offering a more targeted approach to treatment. In comparison to conventional organic or inorganic nanoparticles, DNs offer several advantages: their composition is more transparent and predictable, functionalization is more versatile, and they exhibit improved targeting of specific organelles.^51,63,119−121^ Additionally, DNs have the potential to enhance immunogenicity, allow for more personalized design, and provide a higher level of safety.^51,63,119−121^

**Figure 8:**
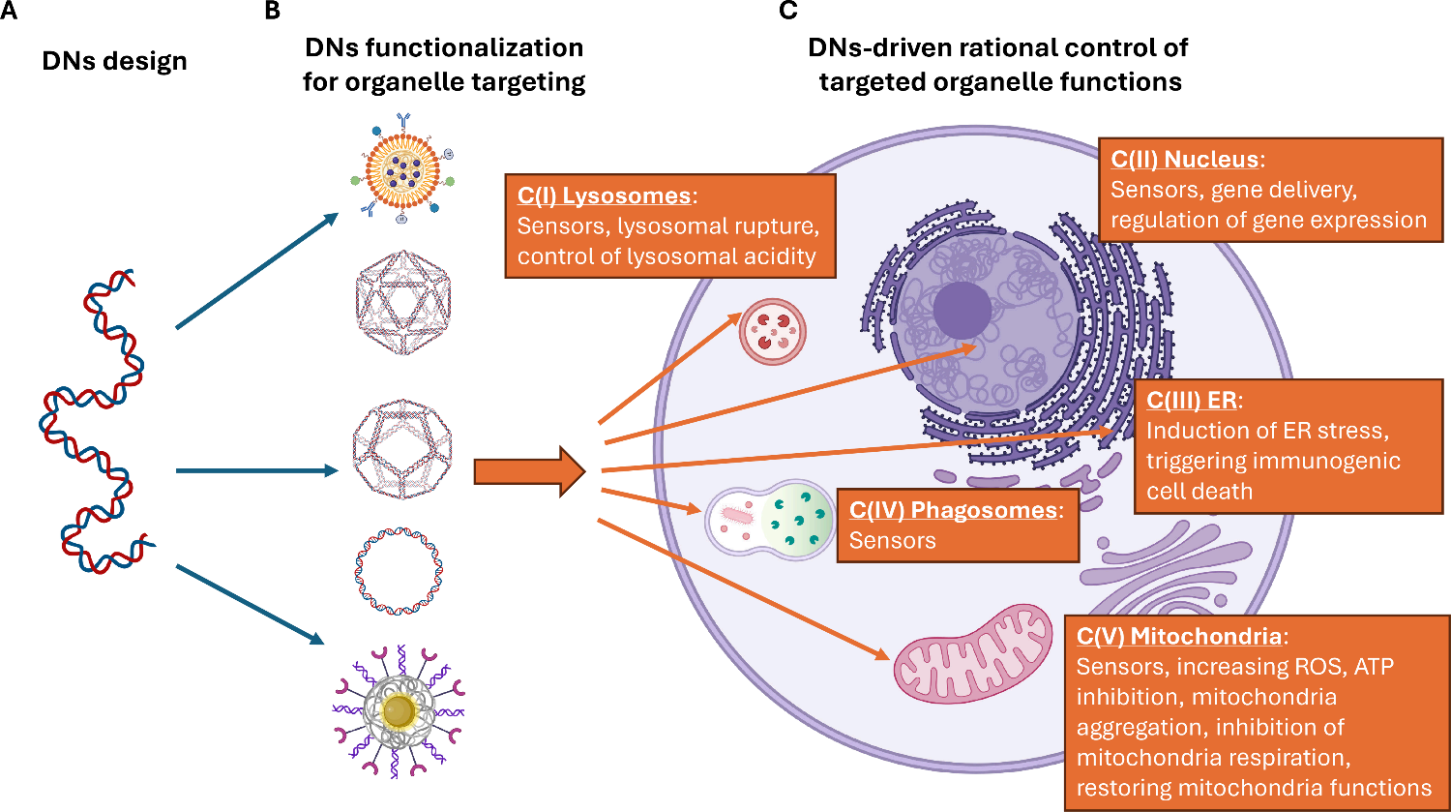
Summary of DN-based organelle targeting. (A) Structural design of DNs is followed by (B) functionalization for specific organelle targeting. (C) Organelle-targeted DNs rationally control cellular functions. C(I) modulation of lysosomal activity via controlling membrane integrity and lysosomal acidity; C(II) gene delivery and regulation of gene expression by nucleus-targeted DNs; C(III) induction of ER stress and activation of immunogenic cell death; C(IV) assessing phagosomal activity by utilizing DNs as sensors; C(V) assessing and controlled manipulation of mitochondria functions. Created with BioRender.

We believe that this Perspective demonstrates the potential of DNs to specifically target different organelles in living cells. We propose that these capabilities enable the rational design and synthesis of DNs that can precisely modulate organelle functions, ultimately controlling cellular activities and physiological outcomes. In fact, the current studies discussed in this Perspective highlight that DNs can be rationally designed to modulate key functions of the nucleus, mitochondria, lysosomes, ER, and plasma membrane (Figure 8). The development of DNs for targeted organelle delivery begins with the careful design of the DNA framework itself. This initial step involves meticulous considerations of the nanostructure’s size, shape, and overall architecture, which are critical for ensuring stability and effective cellular interaction (Figure 8). Once the structural design is finalized, the next step involves functionalizing the DNA nanostructure with specific moieties or ligands. These functional groups are strategically chosen to bind to molecular markers or receptors that are unique to the target organelle, enabling precise and efficient delivery to the desired cellular compartment (Figure 8). However, we would like to conclude by highlighting several challenges from the cell biological perspective, and discussing potential future directions for research aimed at optimizing the organelle-targeting capabilities of DNs and enhancing their ability to rationally modulate organelle functions.

First, as evident from Tables 1 and 2, the majority of current research focuses on the controlled regulation of mitochondrial, lysosomal, and nuclear functions. In contrast, studies targeting other organelles remain underrepresented. We believe that DNs could hold significant promise for targeting ER, Golgi apparatus, and other organelles, presenting exciting opportunities for future exploration. Organelles play a crucial role in regulating cell functions and activity.^48^ Their dysfunction can lead to the development of various pathological conditions and diseases, including cancer, cardiovascular disorders, metabolic syndromes, and neurodegenerative diseases.^48^ For example, dysfunctional mitochondria are associated with tumor progression and metastasis.^153,179^ Lysosomal dysfunction contributes to the development of neurodegenerative diseases such as Gaucher, Parkinson’s, and Alzheimer’s.^180^ Similarly, alterations in ER function can result in depression, neurodegeneration, atherosclerosis, liver disease, and cancer.^181,182^ Golgi apparatus dysfunction are associated with development and progression of neurodegenerative diseases, osteoporosis, cancer, liver cirrhosis and cardiomyopathies.^183^ Golgi-targeted nanodrug delivery systems have shown promising applications in treating a range of diseases, including cancer and neurological disorders.^184^ However, several challenges hinder their clinical translation, such as susceptibility to enzymatic degradation, limited cellular uptake, and recognition by immune cells, which can lead to rapid clearance.^26^ Addressing these limitations requires the development of innovative strategies to enhance stability, improve targeted delivery, and evade immune detection.^26^ Furthermore, establishing rigorous methodologies to investigate the behavior and fate of DNs under physiological conditions is essential for optimizing their therapeutic efficacy and unlocking their full clinical potential.^26,185^

Second, advancing our understanding of the interplay between organelle-specific characteristics and the principles guiding DNs construction will be crucial for the development of improved organelle-targeted therapeutic agents. A deeper analysis of how DNs interact with distinct organelles, both structurally and functionally, will pave the way for designing highly effective and selective treatments. Although controlled delivery at the organelle level has already been demonstrated in preclinical studies, its integration into mainstream medical practice and clinical settings remains an ongoing challenge.^48,110,111^ Addressing issues such as large-scale synthesis, biocompatibility, DN stability, and the ability to adapt these nanoplatforms to the complex tumor microenvironment will be essential for fully realizing their therapeutic potential. Research on the large-scale production of DNs demonstrates significant potential for biotechnological mass production of the DNA strands required for DN assembly, while also lowering the associated production costs.^186^ Furthermore, DNs have the unique potential that multiple structures can be concatenated and/or “nested” together, enabling staged release, or targeting of multiple organelles either simultaneously or sequentially.

Third, the use of primary cell cultures in studying DN-cell interactions remains relatively uncommon (Table 2). Unlike immortalized cell lines, which are genetically modified for continuous passage and *in vitro* subculture, primary cells have a limited lifespan in culture.^187,188^ This finite culture period ensures they retain genomic and phenotypic stability as well as key physiological characteristics throughout their lifespan, making them a more accurate model of native tissue behavior.^187,188^ In contrast, cell lines often undergo significant proteomic and genomic changes due to selective pressures during prolonged passages.^187−189^ Comparative analyses of molecular and functional phenotypes reveal that cell lines tend to redirect cellular resources to prioritize functions associated with proliferation and division.^190^ Cell lines derived from late-stage cancers are especially prone to genetic drift, where repeated passages result in continuous genomic evolution, potentially deviating from the original tumor profile.^187−189^ The preservation of critical features of the tissue or disease of origin is essential for generating meaningful and translational research findings. This is particularly important when studying DN-cell interactions, as the cellular environment can significantly influence nanostructure behavior. Therefore, researchers should carefully consider the use of primary cells over cell lines to ensure more accurate representation of physiological conditions and disease-specific responses.

Fourth, while DNs have shown promising results in *in vivo* studies, their long-term fate and cytotoxic potential remain mostly unknown (Table 2). The efficacy and safety of organelle-targeted treatments using DNs require more comprehensive investigations to fully validate their potential for clinical applications.^51,63,119−121^ Key aspects such as blood clearance rates, retention times at target sites, and physiological interactions with the host system are still insufficiently studied. Without a clear understanding of how DNs behave over extended periods *in vivo*, including their biodistribution, degradation pathways, and possible accumulation in off-target tissues, it is difficult to predict their long-term safety profile.

It is worth noting here, that combining *in vivo* delivery with organelle-targeted delivery presents several challenges in achieving both effective systemic distribution and precise targeting of therapeutics to specific organelles within cells.^48,100,101^ While *in vivo* delivery systems are designed to facilitate broad distribution throughout the body, organelle-targeted systems require a level of specificity that often limits their systemic circulation time.^48,100,101^ For instance, nanoparticles can be engineered to improve their physicochemical properties for better tissue targeting.^191^ However, achieving organelle specificity typically necessitates modifications that can hinder their ability to circulate effectively *in vivo*.^191^ Additionally, targeting specific organelles, such as mitochondria, presents further challenges. The mitochondrion double membrane and negative potential must be overcome to deliver therapeutics effectively.^192^ Lipophilic cations, particularly triphenylphosphonium, have proven to be effective vehicles for mitochondrial delivery due to their ability to permeate cellular membranes and accumulate within mitochondria.^192^ However, successful mitochondrial targeting often requires functionalization with ligands that possess both positive charge and hydrophobicity.^192^ While positively charged materials can enhance targeting, they may also accumulate in organs like the lungs and liver, potentially causing toxicity during *in vivo* delivery.^193^ Surface modifications, such as PEGylation or controlled oxidation, can mitigate this acute toxicity while maintaining delivery efficacy.^194^

A promising approach to address these challenges is the use of dynamic delivery strategies. Dynamic DNs, which undergo environment-responsive structural and functional transformations, can be tailored to exhibit properties suited to different stages of the delivery process. For example, the development of stimulus-responsive DNs capable of responding to biomolecules like nucleic acids and proteins, as well as biophysical environmental factors such as temperature and pH, offers a potential solution for enhancing the precision and efficacy of therapeutic delivery.^195^

Next, physiological monitoring of treated individuals to assess potential side effects or systemic toxicity remains largely unaddressed in current research (Table 2). To overcome these limitations, it is imperative to conduct long-term tracking of DNs to evaluate their safety and therapeutic efficacy. This includes developing advanced *in vivo* experimental models that closely mimic human physiological conditions, such as humanized organoids,^196^ organ-on-a-chip systems,^197,198^ or patient-derived xenografts,^199^ to provide more accurate predictions of clinical outcomes. Additionally, the optimization of DN formulations to enhance biocompatibility, reduce immunogenicity, and achieve controlled degradation will be critical to ensuring their safe translation into clinical settings. Addressing these challenges will be necessary for unlocking the full potential of DNs in next-generation organelle-targeted therapies.

Finally, the exploration of the long-term fate of DNs *in vivo* is closely related to their interaction with the liver and the hepatobiliary system, particularly in terms of metabolization and excretion. However, this critical aspect of DN behavior remains insufficiently studied. Current evidence suggests that the liver can sequester up to 99% of systemically administered nanoparticles, which significantly impacts their biodistribution and therapeutic potential.^17,200−202^ Long-term accumulation of nanoparticles in the liver has been associated with adverse effects, such as hepatotoxicity,^8^ and can significantly limit their clinical efficacy. Drug-induced liver injury (DILI) is a well-documented issue described as an unexpected and harmful impact of drugs on liver function.^203^ DILI represents a serious concern, being one of the leading causes of acute liver failure in Western countries.^203,204^ Moreover, numerous nanoparticles have demonstrated hepatotoxic effects, displaying DILI properties that were often overlooked during initial investigations.^8,17,200−202^ Despite these known challenges, studies explicitly addressing the hepatotoxic potential of DNs are notably absent in the current literature. Understanding and mitigating the hepatotoxicity of DNs is essential for optimizing their design and ensuring their safety in clinical applications. Future research should focus on developing strategies to minimize liver accumulation and off-target effects. This could involve tailoring DN size, charge, surface modifications, and degradation rates to reduce liver sequestration. Additionally, creating advanced *in vitro* and *in vivo* models, such as liver-on-a-chip systems^205,206^ or animal models that mimic human liver metabolism,^207^ could facilitate a deeper understanding of DN-liver interactions. Such studies would enable the rational engineering of clinically viable DNs, ensuring minimal hepatotoxicity while maximizing therapeutic efficacy.

Looking ahead, we believe DN-based organelle targeting research has the potential to revolutionize various therapies at the intersection of biology, nanomaterials, and medicine. Future advancements are likely to focus on the development of smarter construction strategies for DNA-based nanomaterials, enabling them to adapt to and withstand the dynamic chemical changes within complex biological environments. Such innovations will play a pivotal role in advancing precision medicine, offering more targeted, effective, and personalized therapeutic approaches. We are optimiztic that the systematic analysis of current state-of-the-art of DN-based organelle targeting presented in this Perspective will contribute to the ongoing efforts in this pioneering field, inspiring new breakthroughs and fostering collaborative progress toward transformative applications in biomedicine.

## Abbreviations

DNs: DNA nanostructures
FDA: U.S. Food and Drug Administration
EMA: European Medicines Agency
NLS: nuclear localization signal sequence
TAT: transactivator of transcription
ER: endoplasmic reticulum
ITR: inverted-terminal repeat-like
SV40: simian virus 40
DTS: DNA nuclear targeting sequences
Pol II: RNA polymerase II
ROS: reactive oxygen species
APE1: apurinic/apyrimidinic endonuclease 1
SNV: single nucleotide variant
mtDNA: mitochondrial DNA
cGAS-STING: cyclic GMP-AMP synthase-stimulator of interferon genes
AKI: acute kidney injury
PD-L1: programmed cell death ligand-1
HCC: hepatocellular carcinoma
EGFR: epidermal growth factor receptor
ICD: immunogenic cell death
DILI: drug-induced liver injury
